# Supplementary figures and images for: Mitochondrial Protein Carboxyl-Terminal Alanine-Threonine Tailing Promotes Human Glioblastoma Growth by Regulating Mitochondrial Function
Source: bioRxiv. 2026 Jan 9:2024.05.15.594447. Originally published 2024 May 18. Preprint. [Version 5] doi: 10.1101/2024.05.15.594447 (PMC11118334; doi:10.1101/2024.05.15.594447)

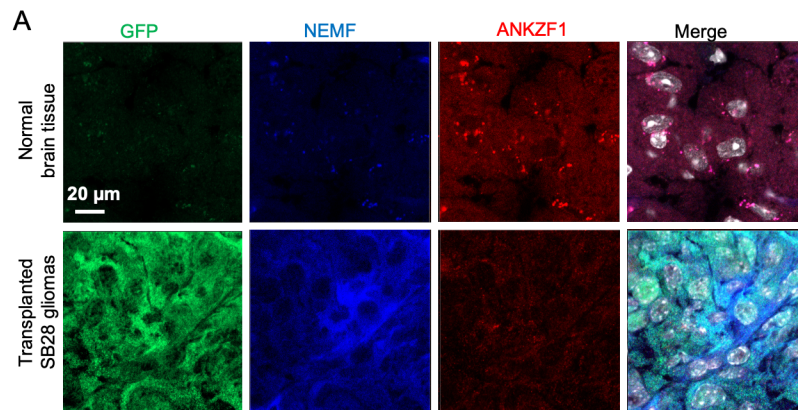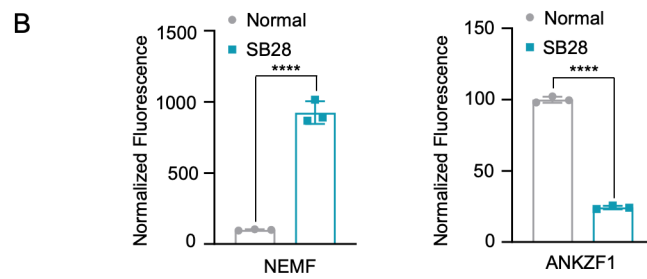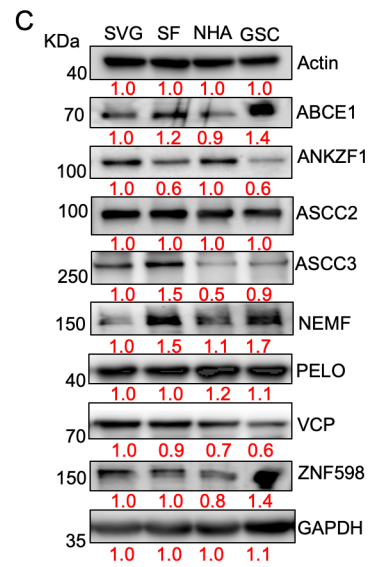

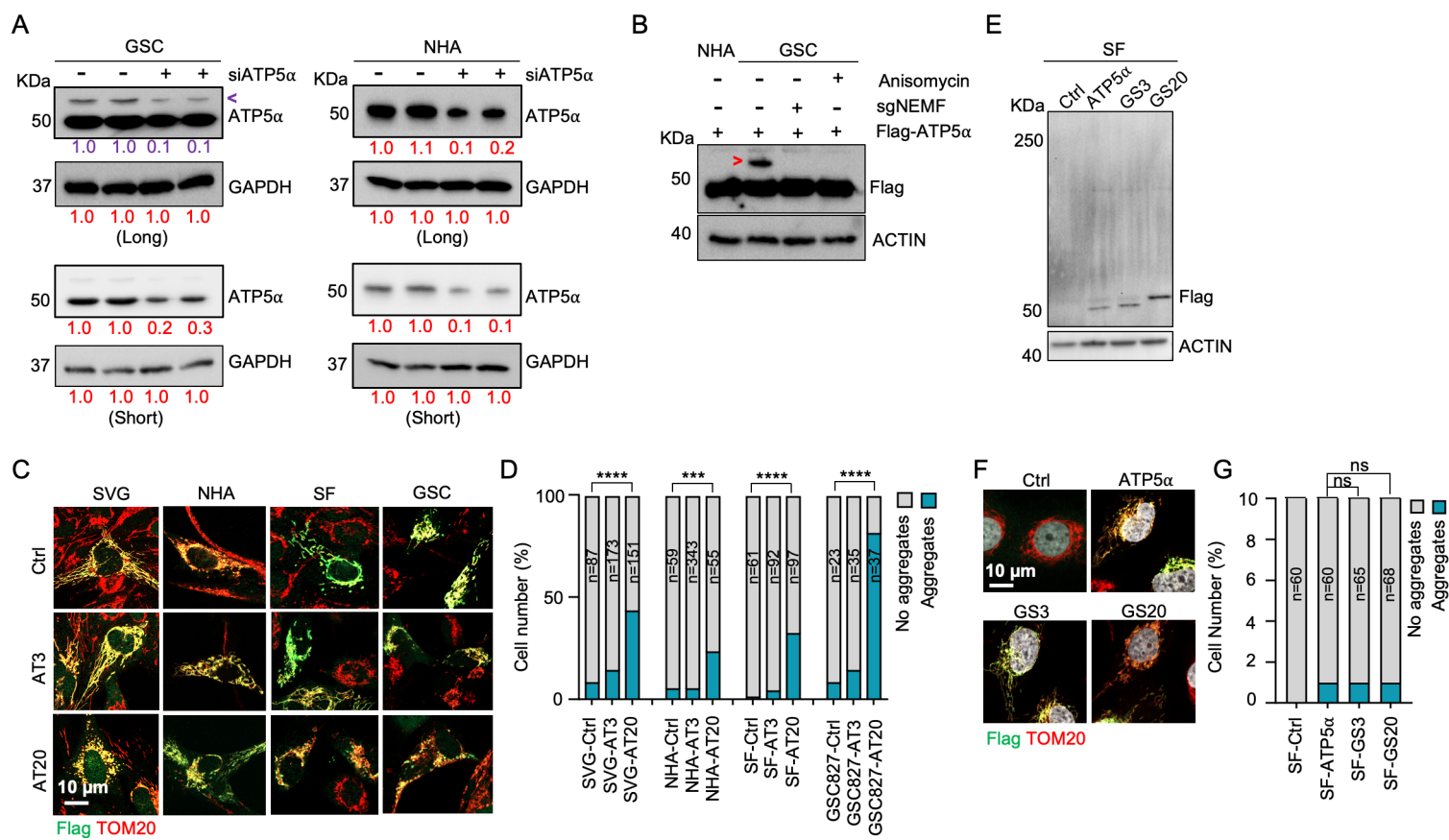

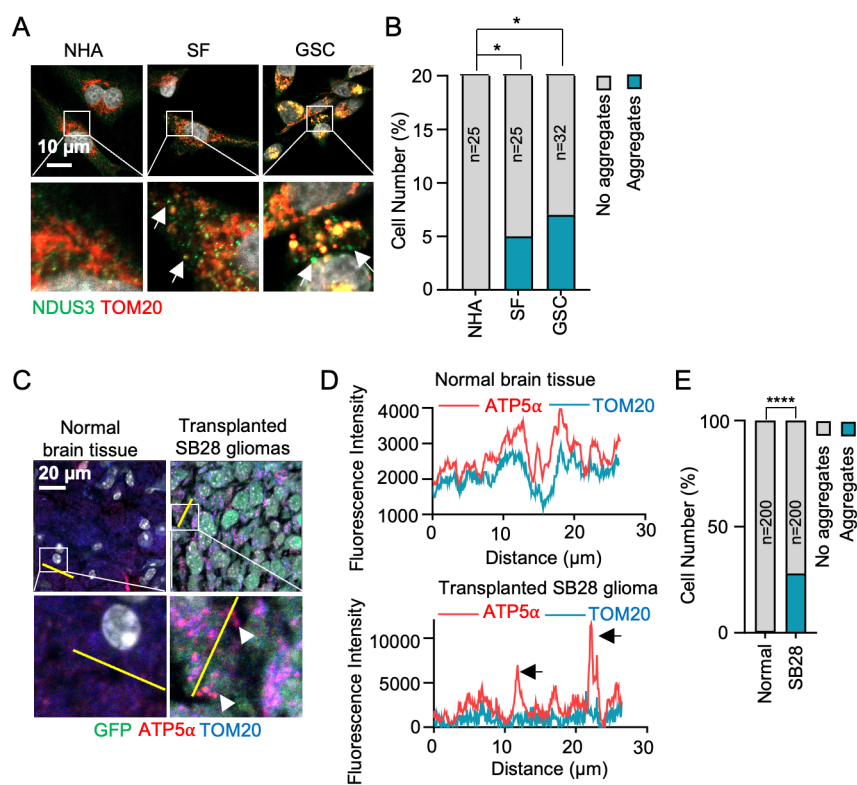

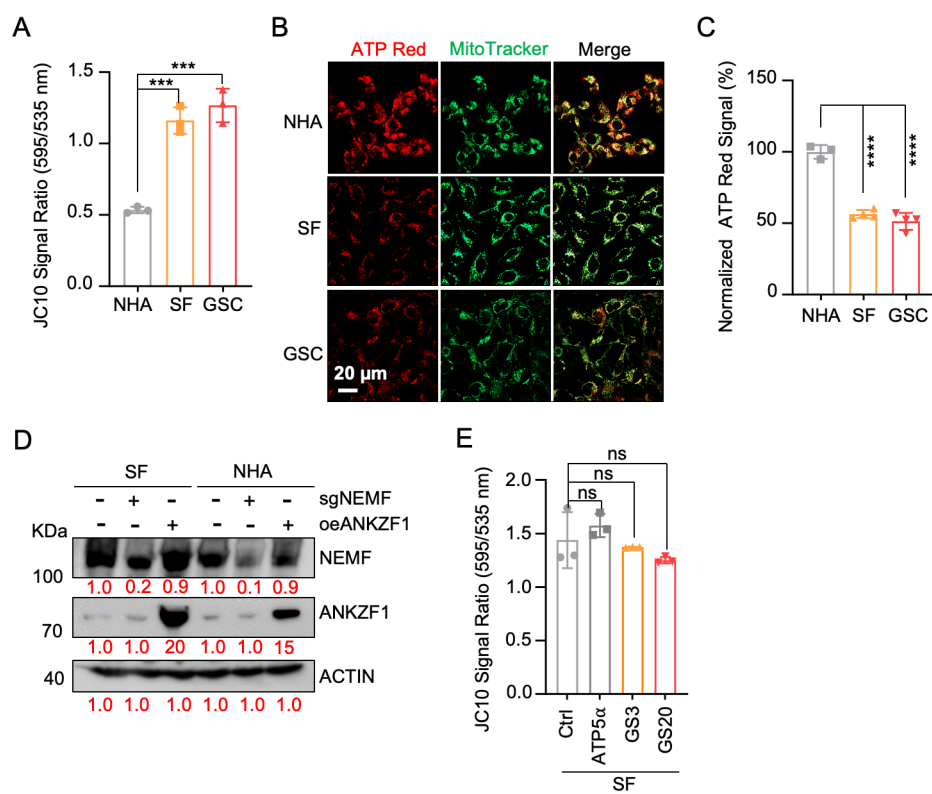

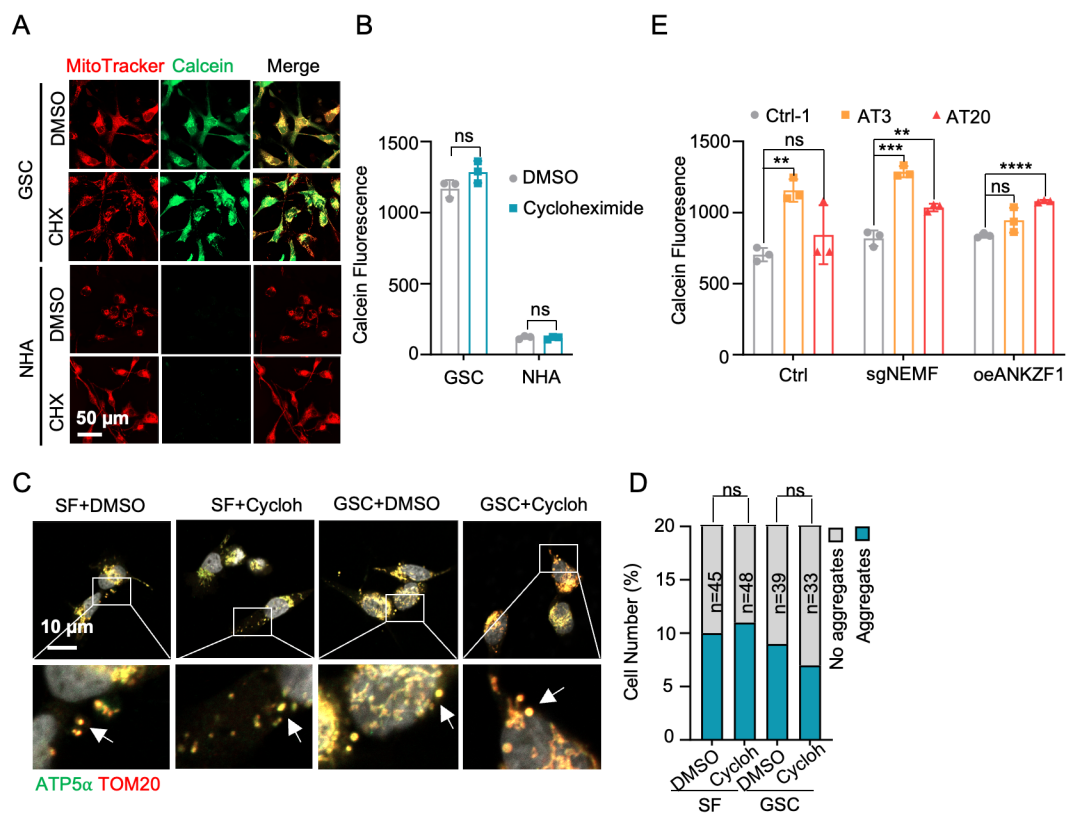

**A**

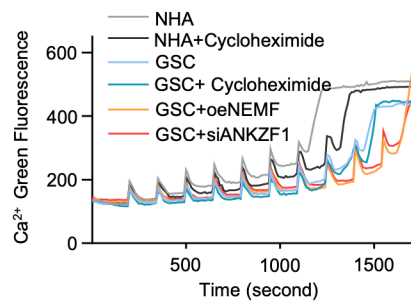

**B**

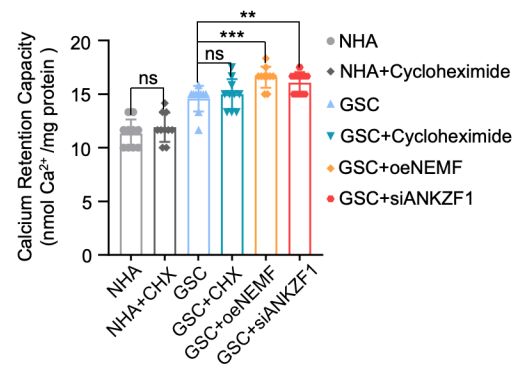

**C**

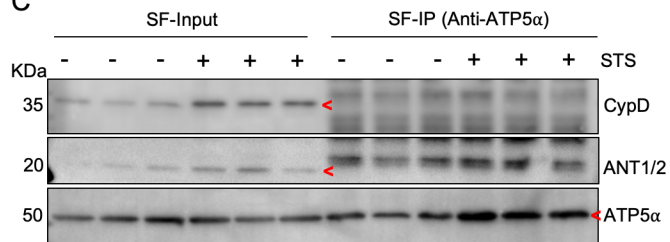

**D**

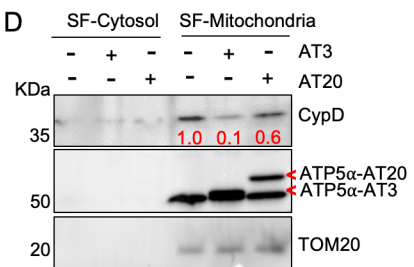

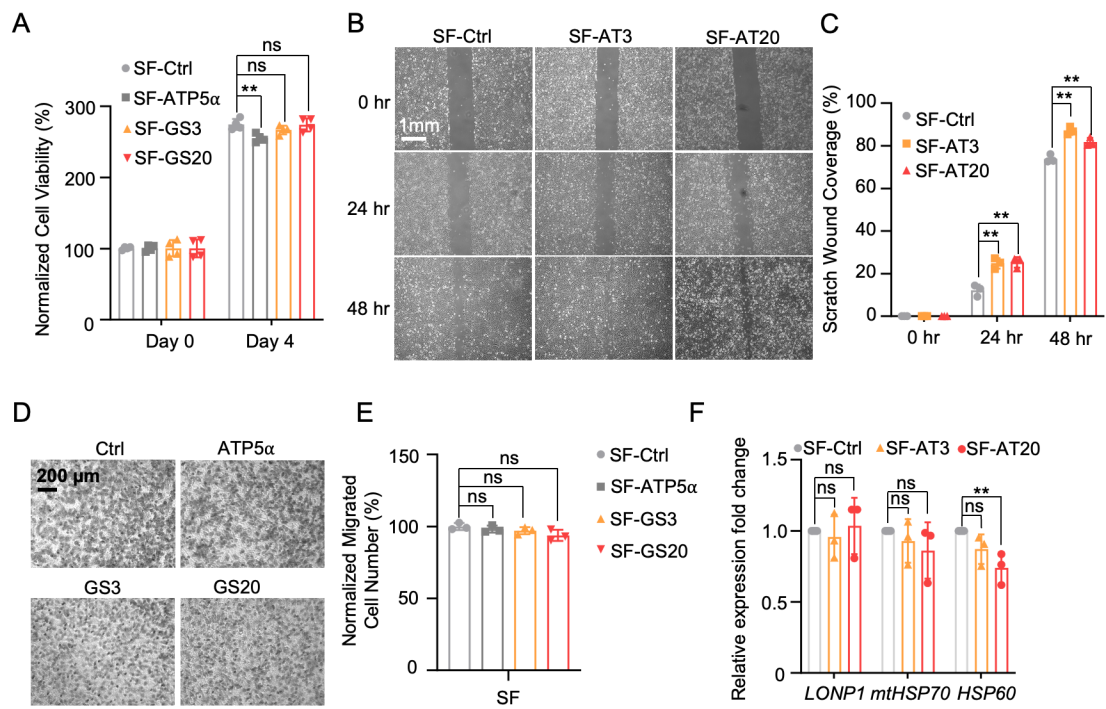

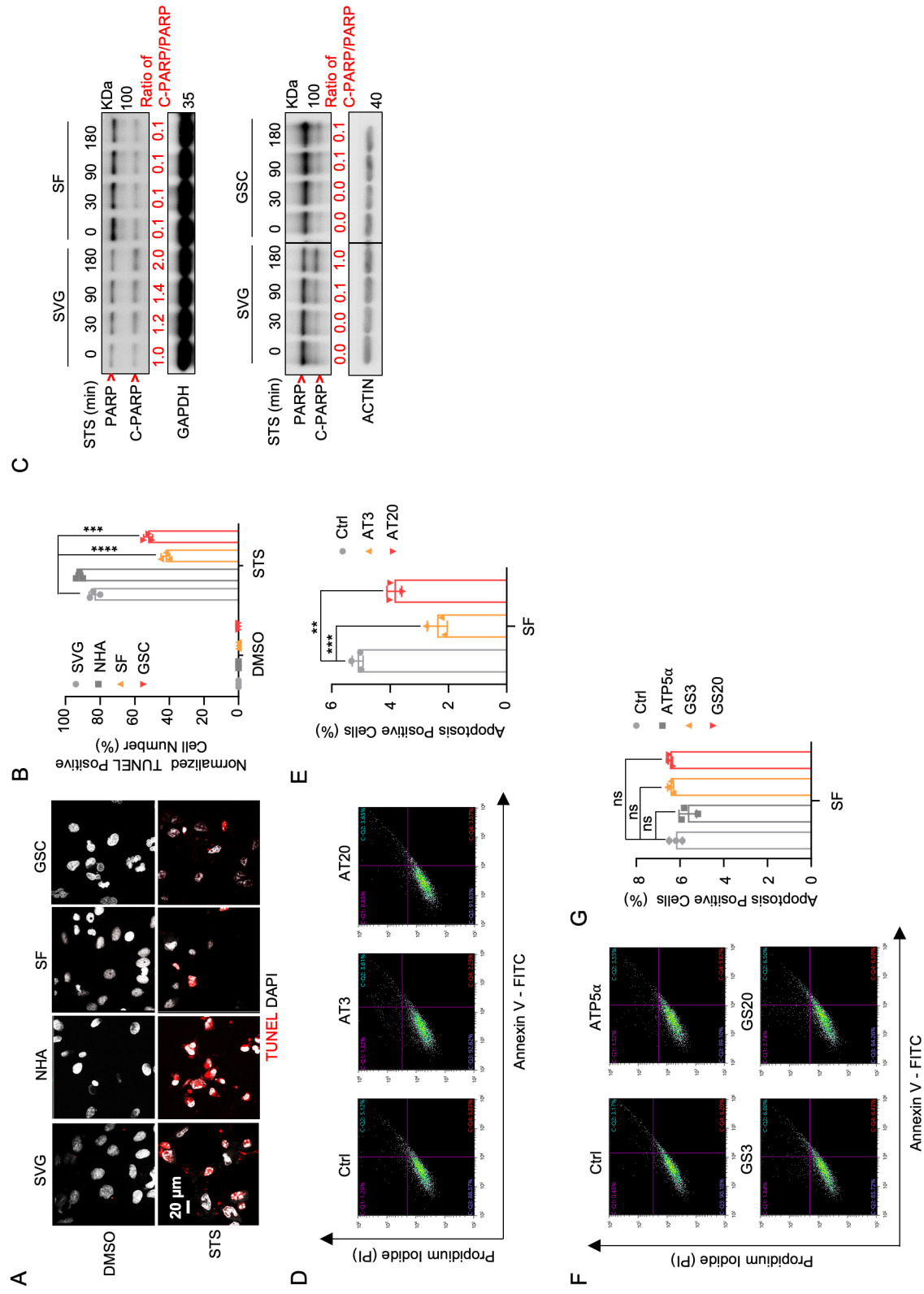

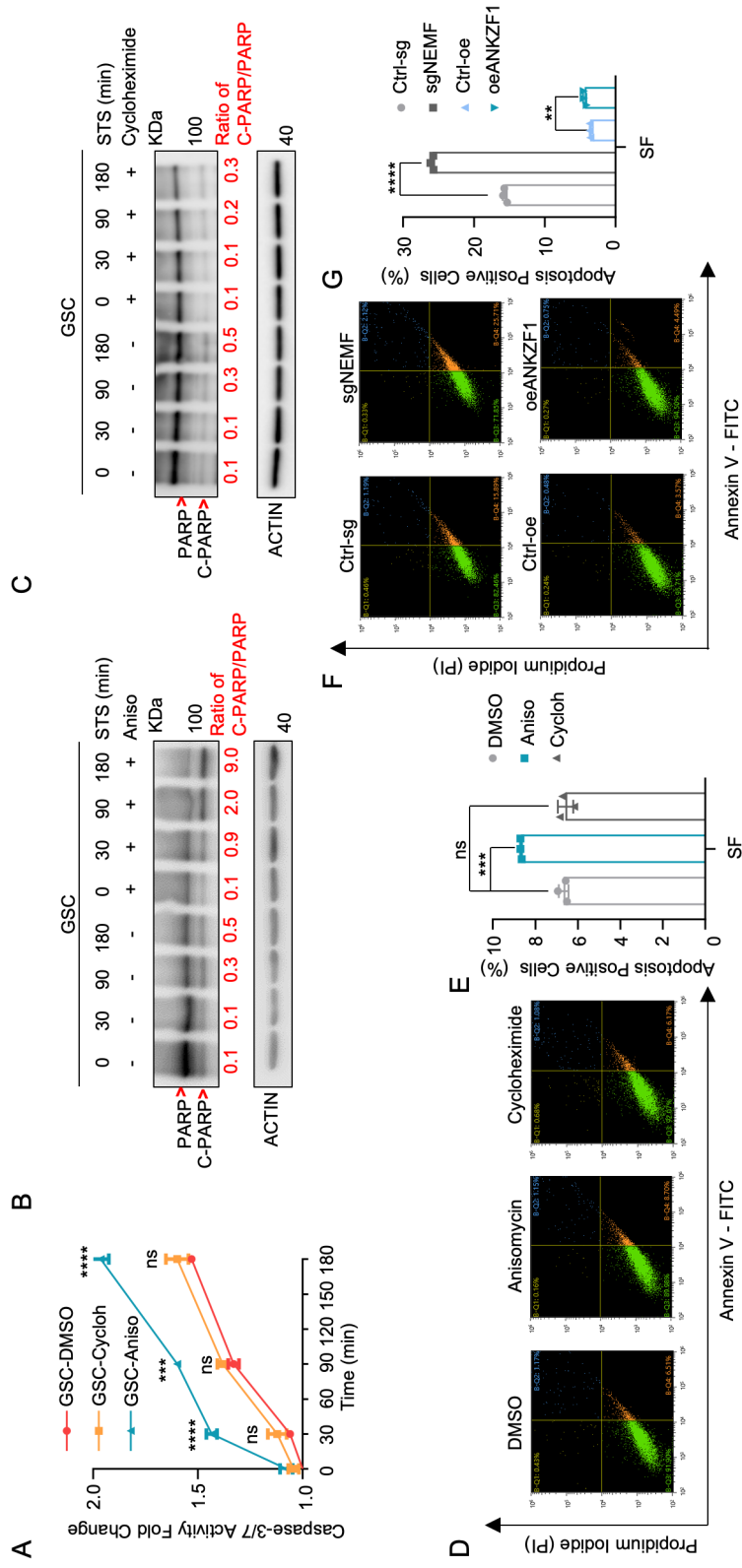

Supplement: Supplement 1 — Figure 1 – Figure Supplement 1. RQC pathway activity in GBM cells (A) Immunofluorescence staining shows elevated NEMF and reduced ANKZF1 endogenous protein levels in the tumor tissue of the GBM mouse model compared to wild-type brain tissue. Tumor identification is indicated by GFP (green). (B) Quantification of A (n=3; unpaired Studenťs t-test; ****, P < 0.0001). (C) Western blot analysis of select RQC factors in control cell lines (SVG, NHA) and GBM cell lines (SF268, GSC827), using ACTIN as the loading control. Red numbers represent fold changes in protein levels relative to controls (SVG). Figure 1 – Figure Supplement 2. AT repeat sequences mimicking CAT-tails induce protein aggregates in cells (A) Western blot analysis of ATP5α in GSC and NHA cells, using GAPDH as the loading control. The purple arrowhead indicates the modified ATP5α form; “short” and “long” refer to exposure time. Red numbers represent fold changes in protein levels relative to controls (the leftmost bands); purple numbers represent fold changes in protein levels of the modified ATP5α form relative to the control (the leftmost band). (B) Western blot analysis of Flag-tagged ATP5α in GSC and control cells, using ACTIN as the loading control. The red arrowhead indicates the modified Flag-ATP5α form. (C) Immunofluorescence staining shows that Flag-tagged ATP5α-AT3 and ATP5α-AT20 (green) form aggregates in GBM and control cells, using TOM20 (red) as a mitochondrial marker. (D) Quantification of C (n=3; chi-squared test; ***, P < 0.001; ****, P < 0.0001); the total number of cells counted is indicated in the columns. (E) Western blot of Flag-tagged ATP5α-GS3 and ATP5α-GS20 in GBM cells, using ACTIN as the loading control. (F) Immunofluorescence staining shows that Flag-tagged ATP5α-GS3 and ATP5α-GS20 (green) do not form aggregates in GBM cells, using TOM20 (red) as a mitochondrial marker. (G) Quantification of F (n=3; chi-squared test; ns, not significant); the total number of cells counted is indi [file media-1.pdf]
