# Supplementary material for: Mitochondrial Protein Carboxyl-Terminal Alanine-Threonine Tailing Promotes Glioblastoma Tumor Growth by Regulating Mitochondrial Function
Source: bioRxiv. 2025 Oct 22:2024.05.15.594447. Originally published 2024 May 18. Preprint. [Version 4] doi: 10.1101/2024.05.15.594447 (PMC11118334; doi:10.1101/2024.05.15.594447)
Supplement: Supplementary file 1 [file media-1.pdf]

Figure S1

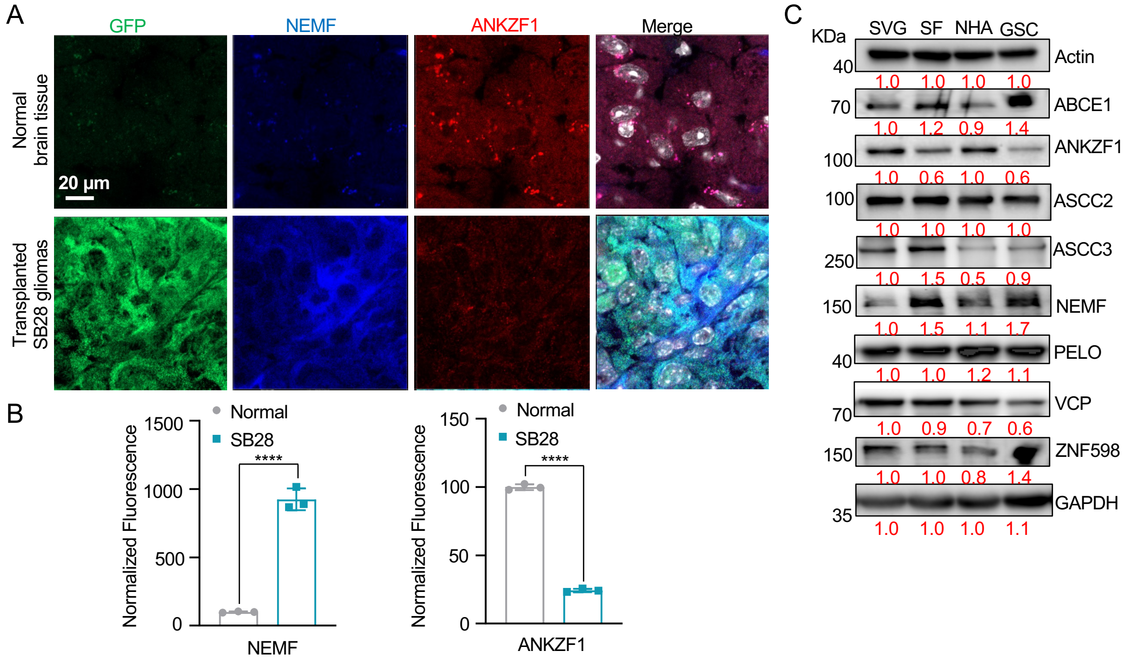
A
GFP
NEMF
ANKZF1
Merge
Normal brain tissue
Transplanted SB28 gliomas
20 μm
B
Normal
SB28
Normalized Fluorescence
1500
1000
500
0
****
NEMF
150
100
50
0
ANKZF1
C
KDa
SVG
SF
NHA
GSC
Actin
ABCE1
ANKZF1
ASCC2
ASCC3
NEMF
PELO
VCP
ZNF598
GAPDH
40
70
100
100
250
150
40
70
150
35
1.0 1.0 1.0 1.0
1.0 1.2 0.9 1.4
1.0 0.6 1.0 0.6
1.0 1.0 1.0 1.0
1.0 1.5 0.5 0.9
1.0 1.5 1.1 1.7
1.0 1.0 1.2 1.1
1.0 0.9 0.7 0.6
1.0 1.0 0.8 1.4
1.0 1.0 1.0 1.1

Figure S2

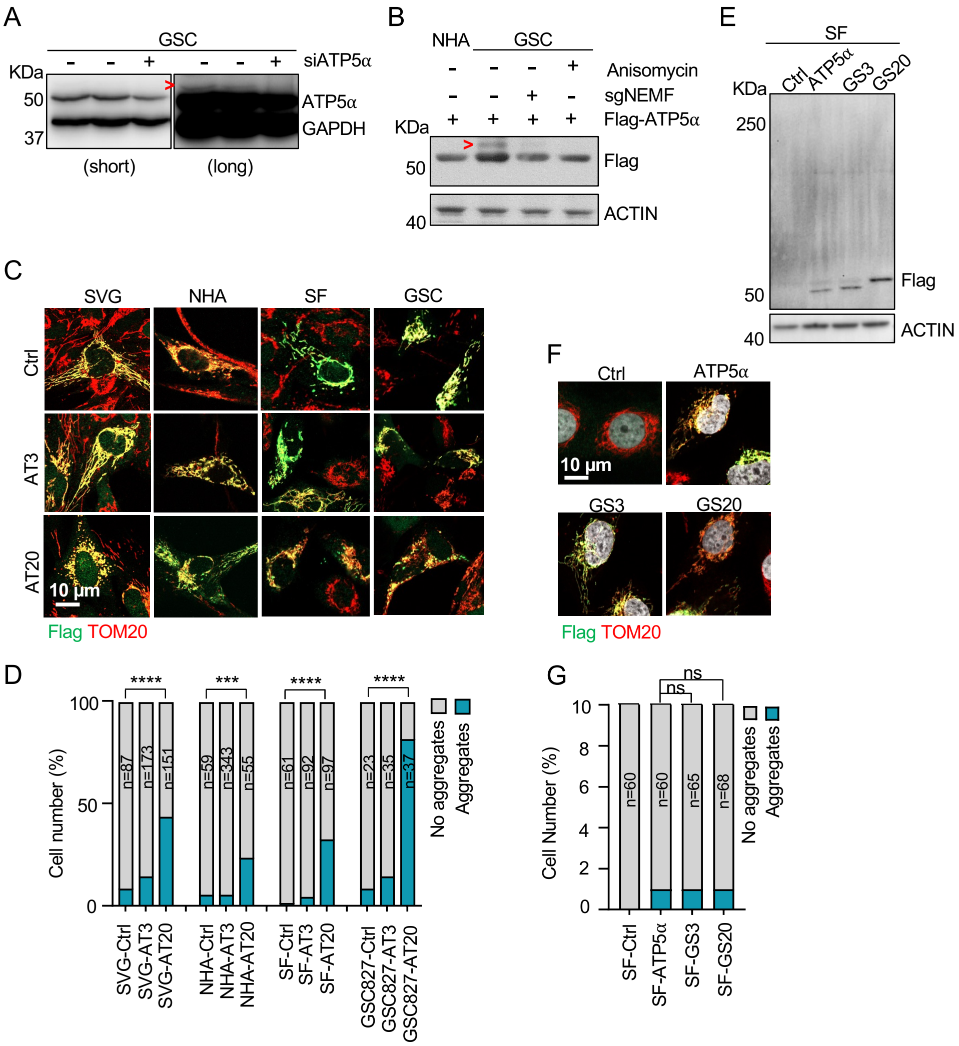
A
GSC
siATP5α
KDa
50
37
ATP5α
GAPDH
(short)
(long)
B
NHA
GSC
Anisomycin
sgNEMF
Flag-ATP5α
KDa
50
40
Flag
ACTIN
E
SF
KDa
Ctrl
ATP5α
GS3
GS20
250
50
40
Flag
ACTIN
C
SVG
NHA
SF
GSC
Ctrl
AT3
AT20
10 μm
Flag TOM20
F
Ctrl
ATP5α
10 μm
GS3
GS20
Flag TOM20
D
Cell number (%)
100
50
0
****
***
****
****
n=87
n=173
n=151
n=59
n=343
n=55
n=61
n=92
n=97
n=23
n=35
n=37
No aggregates
Aggregates
SVG-Ctrl
SVG-AT3
SVG-AT20
NHA-Ctrl
NHA-AT3
NHA-AT20
SF-Ctrl
SF-AT3
SF-AT20
GSC827-Ctrl
GSC827-AT3
GSC827-AT20
G
Cell Number (%)
10
8
6
4
2
0
ns
ns
n=60
n=60
n=65
n=68
No aggregates
Aggregates
SF-Ctrl
SF-ATP5α
SF-GS3
SF-GS20

Figure S3

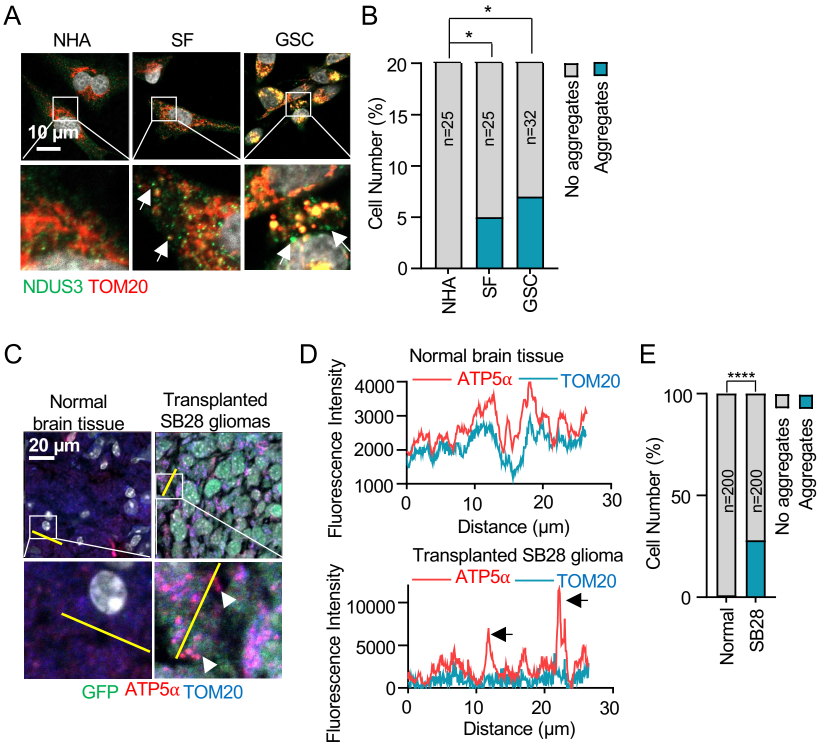

A
NHA
SF
GSC
10 μm
NDUS3 TOM20
B
Cell Number (%)
n=25
n=25
n=32
NHA
SF
GSC
No aggregates
Aggregates
C
Normal brain tissue
Transplanted SB28 gliomas
20 μm
GFP ATP5α TOM20
D
Normal brain tissue
ATP5α
TOM20
Fluorescence Intensity
Distance (μm)
Transplanted SB28 glioma
E
Cell Number (%)
n=200
n=200
Normal
SB28
No aggregates
Aggregates

Figure S4

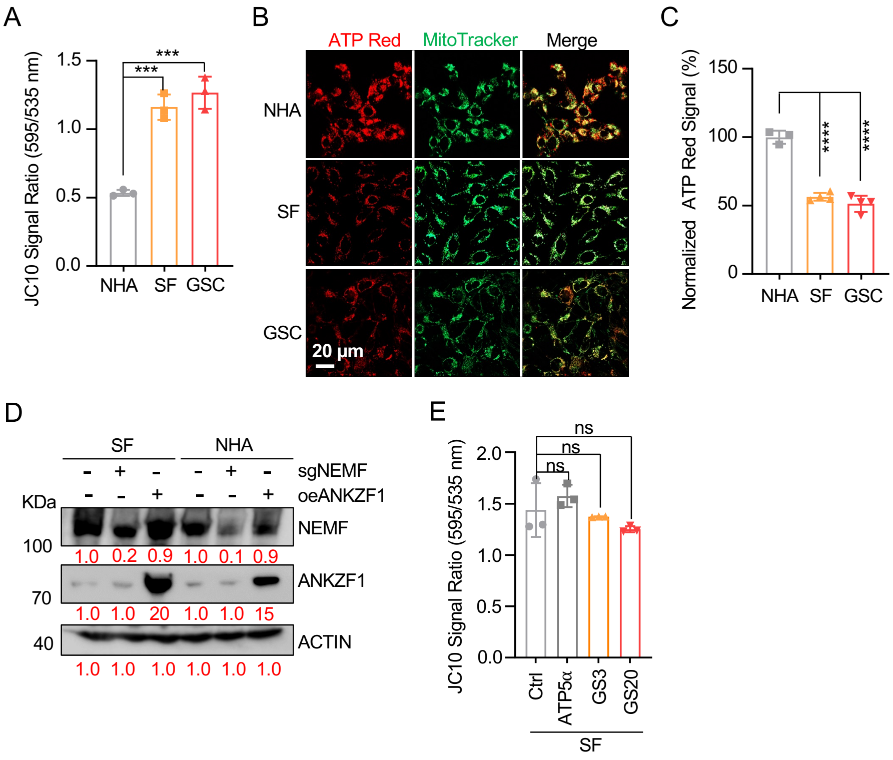

A
JC10 Signal Ratio (595/535 nm)
1.5
1.0
0.5
0.0
***
***
NHA
SF
GSC
B
ATP Red
MitoTracker
Merge
NHA
SF
GSC
20 μm
C
Normalized ATP Red Signal (%)
150
100
50
0
****
****
NHA
SF
GSC
D
SF
NHA
sgNEMF
oeANKZF1
KDa
NEMF
100
1.0 0.2 0.9 1.0 0.1 0.9
ANKZF1
70
1.0 1.0 20 1.0 1.0 15
ACTIN
40
1.0 1.0 1.0 1.0 1.0 1.0
E
JC10 Signal Ratio (595/535 nm)
2.0
1.5
1.0
0.5
0.0
ns
ns
ns
Ctrl
ATP5α
GS3
GS20
SF

Figure S5

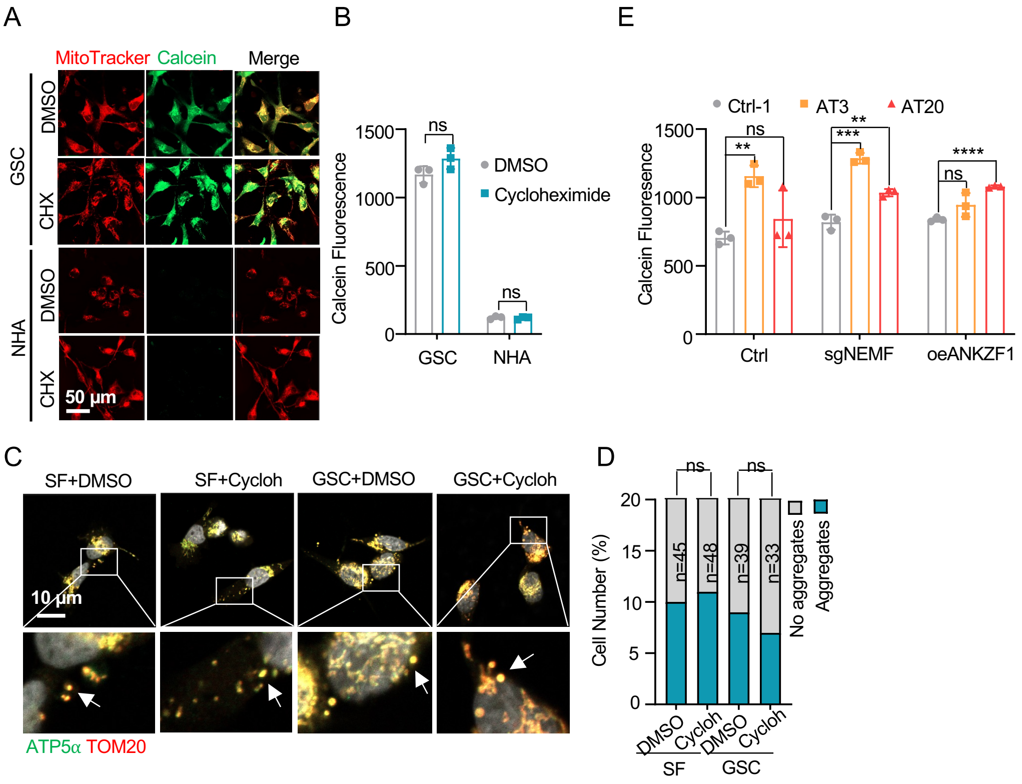

A
MitoTracker
Calcein
Merge
GSC
DMSO
CHX
NHA
DMSO
CHX
50 µm
B
Calcein Fluorescence
1500
1000
500
0
ns
ns
DMSO
Cycloheximide
GSC
NHA
E
Ctrl-1
AT3
AT20
Calcein Fluoresence
1500
1000
500
0
ns
**
**
***
ns
****
Ctrl
sgNEMF
oeANKZF1
C
SF+DMSO
SF+Cycloh
GSC+DMSO
GSC+Cycloh
10 µm
ATP5α TOM20
D
Cell Number (%)
20
15
10
5
0
ns
ns
n=45
n=48
n=39
n=33
No aggregates
Aggregates
DMSO
Cycloh
DMSO
Cycloh
SF
GSC

Figure S6

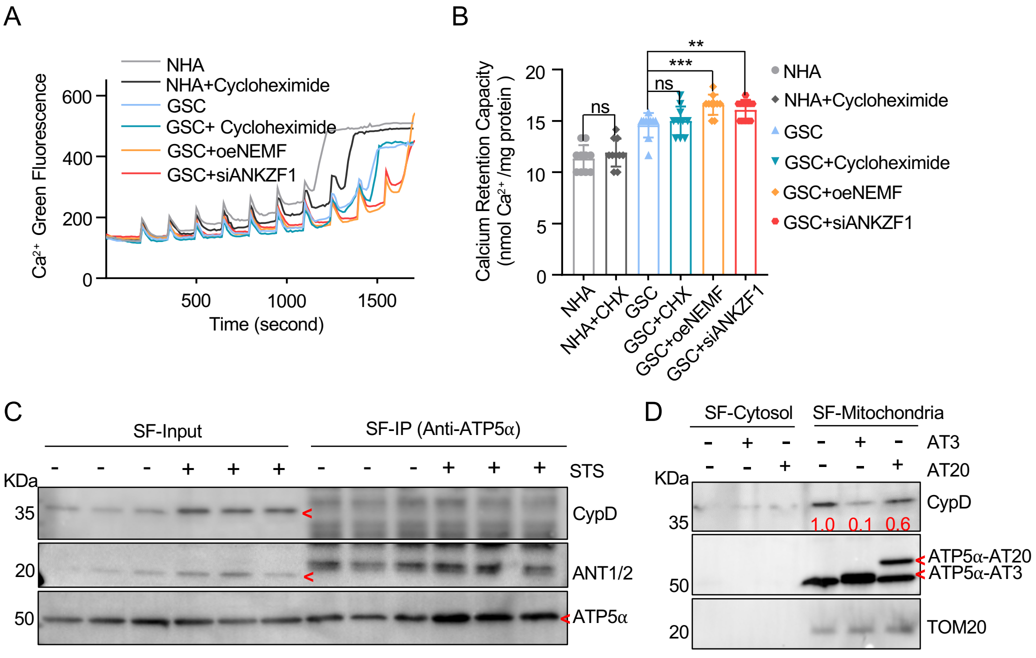
A
NHA
NHA+Cycloheximide
GSC
GSC+ Cycloheximide
GSC+oeNEMF
GSC+siANKZF1
Ca2+ Green Fluorescence
Time (second)
B
Calcium Retention Capacity (nmol Ca2+ /mg protein)
ns
***
**
NHA
NHA+CHX
GSC
GSC+CHX
GSC+oeNEMF
GSC+siANKZF1
NHA
NHA+Cycloheximide
GSC
GSC+Cycloheximide
GSC+oeNEMF
GSC+siANKZF1
C
SF-Input
SF-IP (Anti-ATP5α)
STS
KDa
CypD
ANT1/2
ATP5α
D
SF-Cytosol
SF-Mitochondria
AT3
AT20
KDa
CypD
1.0 0.1 0.6
ATP5α-AT20
ATP5α-AT3
TOM20

Figure S7

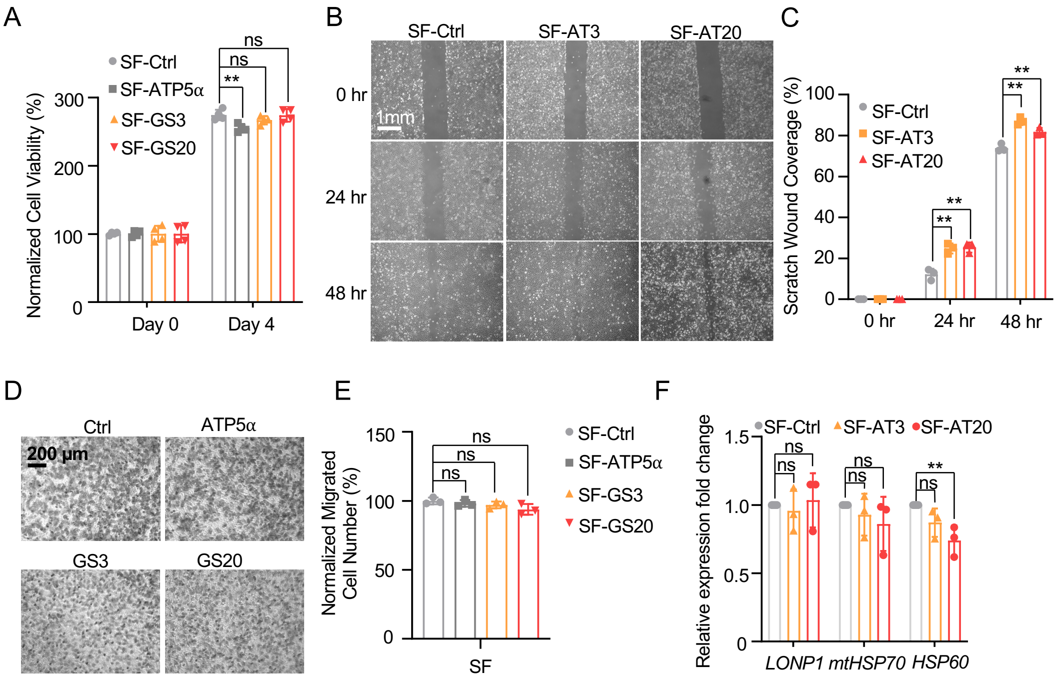

A
SF-Ctrl
SF-ATP5α
SF-GS3
SF-GS20
Normalized Cell Viability (%)
Day 0
Day 4
B
SF-Ctrl
SF-AT3
SF-AT20
0 hr
24 hr
48 hr
1mm
C
Scratch Wound Coverage (%)
SF-Ctrl
SF-AT3
SF-AT20
0 hr
24 hr
48 hr
D
Ctrl
ATP5α
GS3
GS20
200 μm
E
Normalized Migrated Cell Number (%)
SF-Ctrl
SF-ATP5α
SF-GS3
SF-GS20
SF
F
Relative expression fold change
SF-Ctrl
SF-AT3
SF-AT20
LONP1
mtHSP70
HSP60

Figure S8

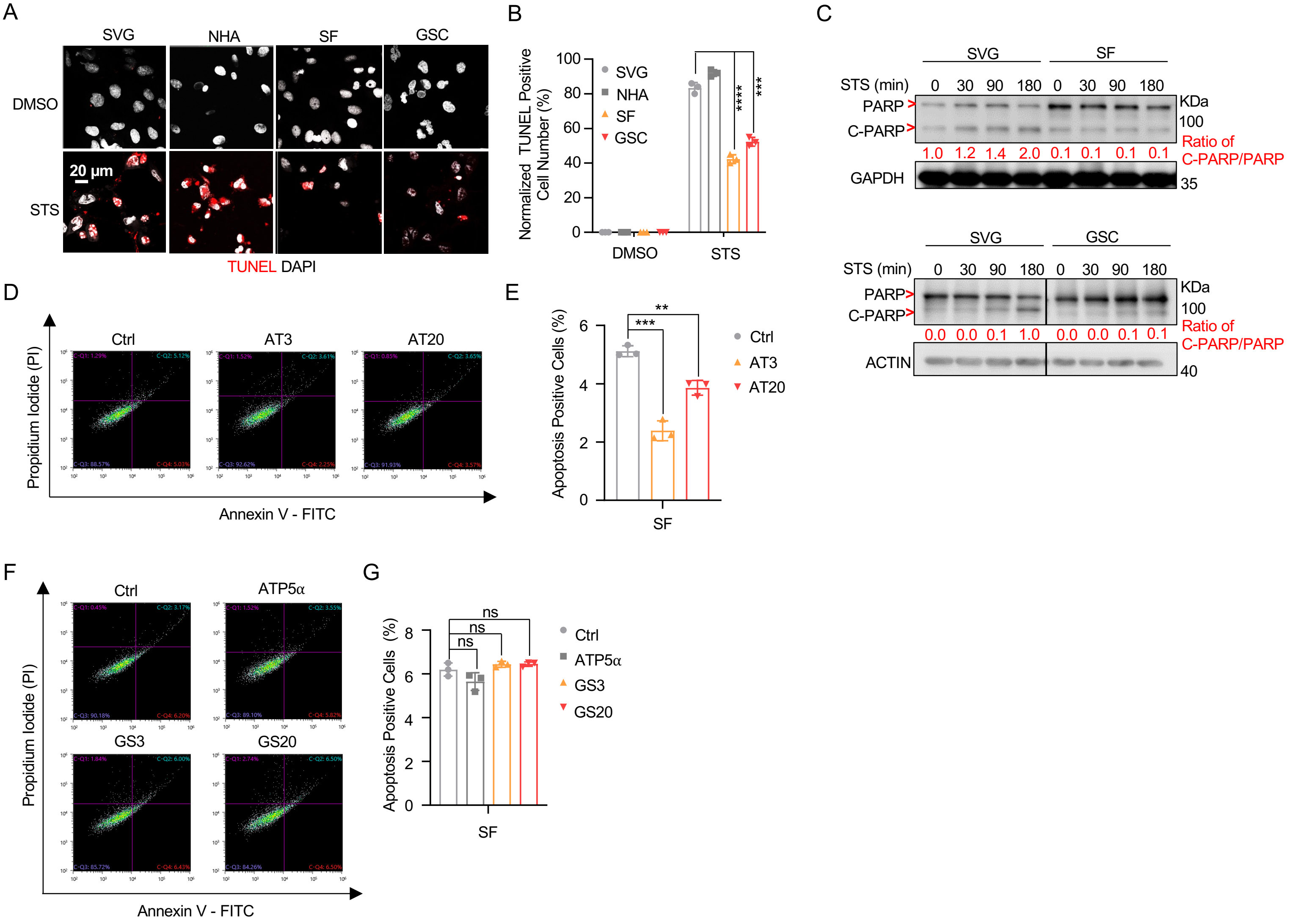

A
SVG
NHA
SF
GSC
DMSO
STS
20 µm
TUNEL DAPI
B
Normalized TUNEL Positive Cell Number (%)
SVG
NHA
SF
GSC
DMSO
STS
****
***
C
SVG
SF
STS (min) 0 30 90 180 0 30 90 180
PARP
C-PARP
KDa
100
Ratio of C-PARP/PARP
1.0 1.2 1.4 2.0 0.1 0.1 0.1 0.1
GAPDH
35
SVG
GSC
STS (min) 0 30 90 180 0 30 90 180
PARP
C-PARP
KDa
100
Ratio of C-PARP/PARP
0.0 0.0 0.1 1.0 0.0 0.0 0.1 0.1
ACTIN
40
D
Ctrl
AT3
AT20
Propidium Iodide (PI)
Annexin V - FITC
E
Apoptosis Positive Cells (%)
Ctrl
AT3
AT20
***
**
SF
F
Ctrl
ATP5α
GS3
GS20
Propidium Iodide (PI)
Annexin V - FITC
G
Apoptosis Positive Cells (%)
ns
ns
ns
Ctrl
ATP5α
GS3
GS20
SF

Figure S9

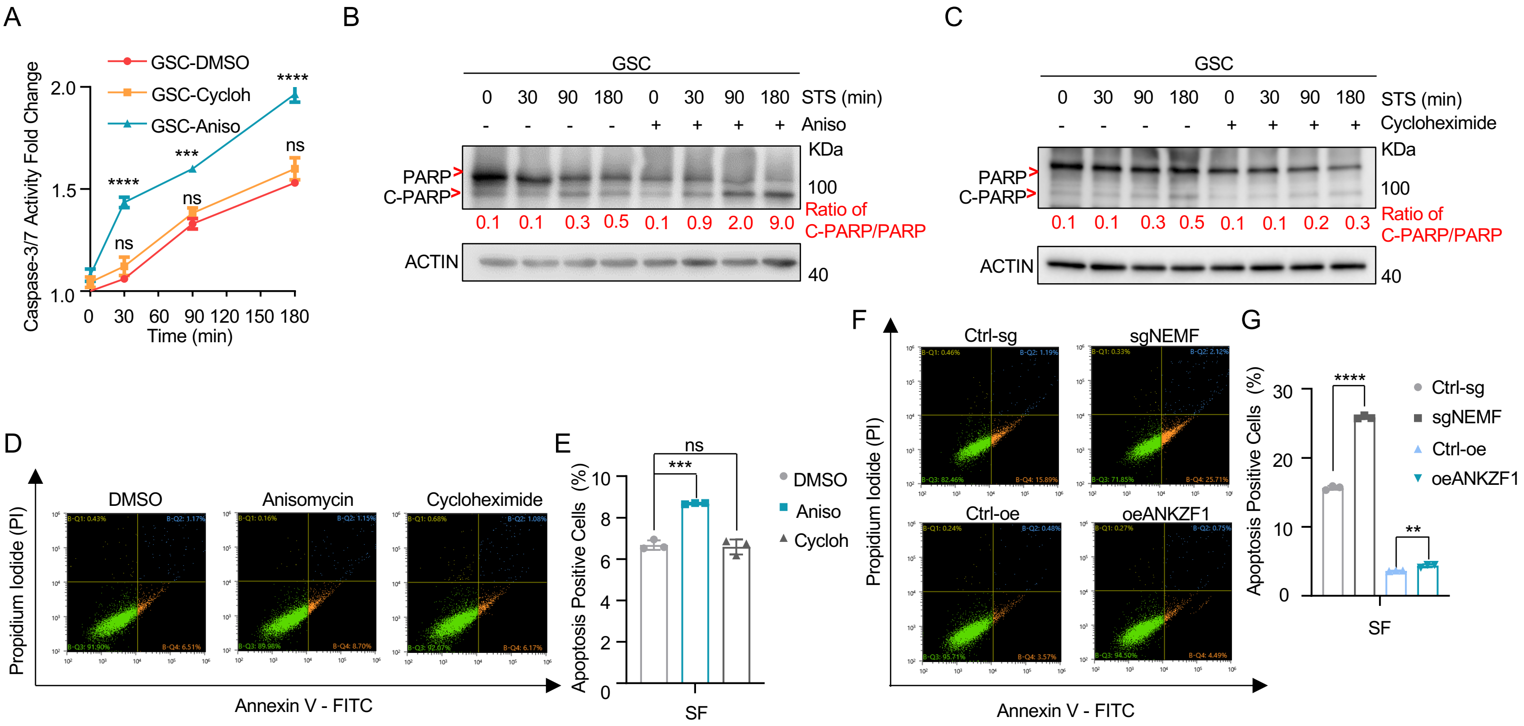

A
GSC-DMSO
GSC-Cycloh
GSC-Aniso
Caspase-3/7 Activity Fold Change
Time (min)
****
***
ns
B
GSC
0 30 90 180 0 30 90 180 STS (min)
- - - - + + + + Aniso
KDa
PARP
C-PARP
100
0.1 0.1 0.3 0.5 0.1 0.9 2.0 9.0
Ratio of C-PARP/PARP
ACTIN
40
C
GSC
0 30 90 180 0 30 90 180 STS (min)
- - - - + + + + Cycloheximide
KDa
PARP
C-PARP
100
0.1 0.1 0.3 0.5 0.1 0.1 0.2 0.3
Ratio of C-PARP/PARP
ACTIN
40
D
DMSO
Anisomycin
Cycloheximide
Propidium Iodide (PI)
Annexin V - FITC
E
Apoptosis Positive Cells (%)
DMSO
Aniso
Cycloh
SF
F
Ctrl-sg
sgNEMF
Ctrl-oe
oeANKZF1
Propidium Iodide (PI)
Annexin V - FITC
G
Apoptosis Positive Cells (%)
Ctrl-sg
sgNEMF
Ctrl-oe
oeANKZF1
SF

Table S1

Table S1. Differential expression analysis for RQC genes in GBM patients and healthy controls

Gene	logFC	AveExpr	t	P.Value	adj.P.Val
RACK1 ⬆	2.224548565	9.048113333	19.24641934	2.62E-57	6.69E-56
ASCC3 ⬆	1.738216567	2.717246389	19.27211713	2.05E-57	5.27E-56
ASCC1 ⬆	1.689584768	4.257915556	17.8774401	1.20E-51	2.18E-50
ASCC2 ⬆	1.471467207	4.153399167	15.9118075	1.43E-43	1.68E-42
ABCE1 ⬆	1.32826428	3.81695	13.79248369	4.69E-35	3.60E-34
VCP ⬆	1.050066326	6.321021667	9.828944092	2.33E-20	9.31E-20
GIGYF2	0.985695112	3.786421389	10.25440005	8.00E-22	3.41E-21
MAP3K20	0.962218073	1.863793889	8.711292467	1.10E-16	3.75E-16
PELO	0.92860628	2.2885625	10.60122263	4.85E-23	2.18E-22
KLHDC10	0.854921284	3.492322222	9.064976509	8.08E-18	2.88E-17
EDF1	0.82091202	8.444505	7.278600017	2.12E-12	5.94E-12
XRN1	0.809119864	1.518371111	8.54020086	3.80E-16	1.26E-15
LTN1	0.786409776	1.9716	9.962815742	8.14E-21	3.32E-20
MKRN1	0.769369764	5.745359444	7.400071791	9.65E-13	2.74E-12
RCHY1	0.652647968	4.276126111	6.840213538	3.40E-11	8.93E-11
ZNF598	0.62380412	4.006663611	6.043582081	3.76E-09	8.90E-09
HBS1L	0.291107388	4.701389722	2.72853772	0.006672805	0.010370549
NEMF	0.194631373	4.566962778	2.575063266	0.010419695	0.015855894
ANKZF1 ⬇	-0.436070986	4.620298333	-3.65005718	0.000300886	0.000525859

Abbreviations: RQC, ribosome-associated quality control; GBM, glioblastoma.
