# Supplementary material for: Mitochondrial stress-induced protein carboxyl-terminal alanine threonine tailing (msiCAT-tailing) facilitates glioblastoma tumorigenesis through the modulation of mitochondrial functions
Source: bioRxiv. 2025 Sep 20:2024.05.15.594447. Originally published 2024 May 18. Preprint. [Version 3] doi: 10.1101/2024.05.15.594447 (PMC11118334; doi:10.1101/2024.05.15.594447)
Supplement: Supplementary file 1 [file media-1.pdf]

# Figure S1

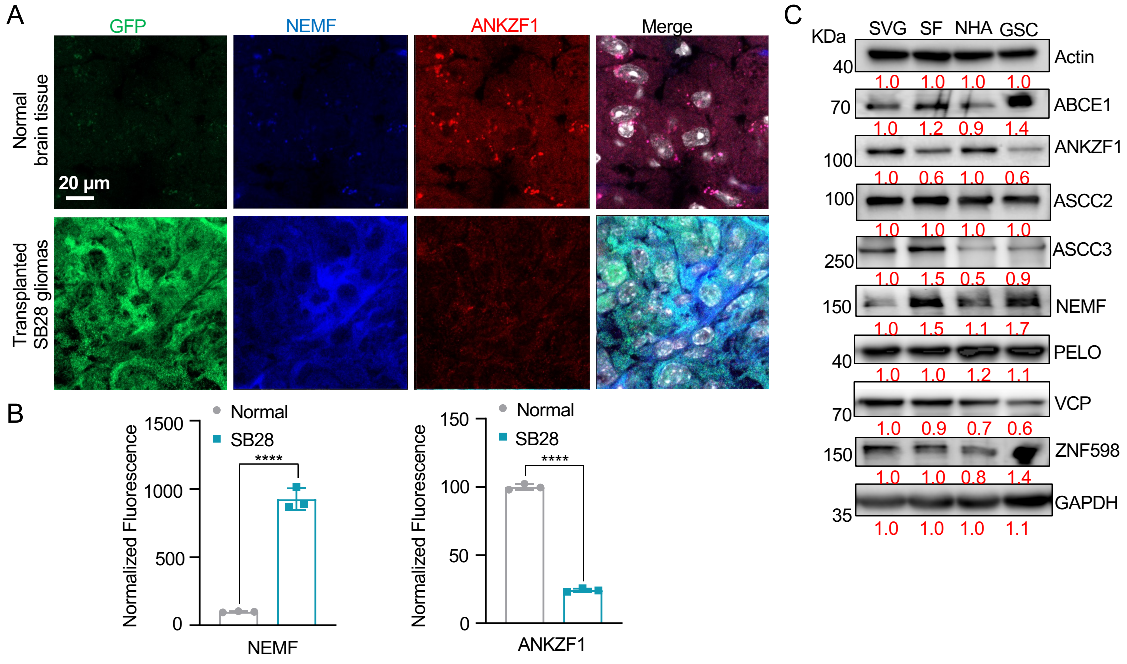
A
GFP
NEMF
ANKZF1
Merge
Normal brain tissue
Transplanted SB28 gliomas
20 µm
B
Normal
SB28
****
1500
1000
500
0
Normalized Fluorescence
NEMF
150
100
50
0
ANKZF1
C
KDa
SVG
SF
NHA
GSC
Actin
ABCE1
ANKZF1
ASCC2
ASCC3
NEMF
PELO
VCP
ZNF598
GAPDH
40
70
100
100
250
150
40
70
150
35
1.0 1.0 1.0 1.0
1.0 1.2 0.9 1.4
1.0 0.6 1.0 0.6
1.0 1.0 1.0 1.0
1.0 1.5 0.5 0.9
1.0 1.5 1.1 1.7
1.0 1.0 1.2 1.1
1.0 0.9 0.7 0.6
1.0 1.0 0.8 1.4
1.0 1.0 1.0 1.1

# Figure S2

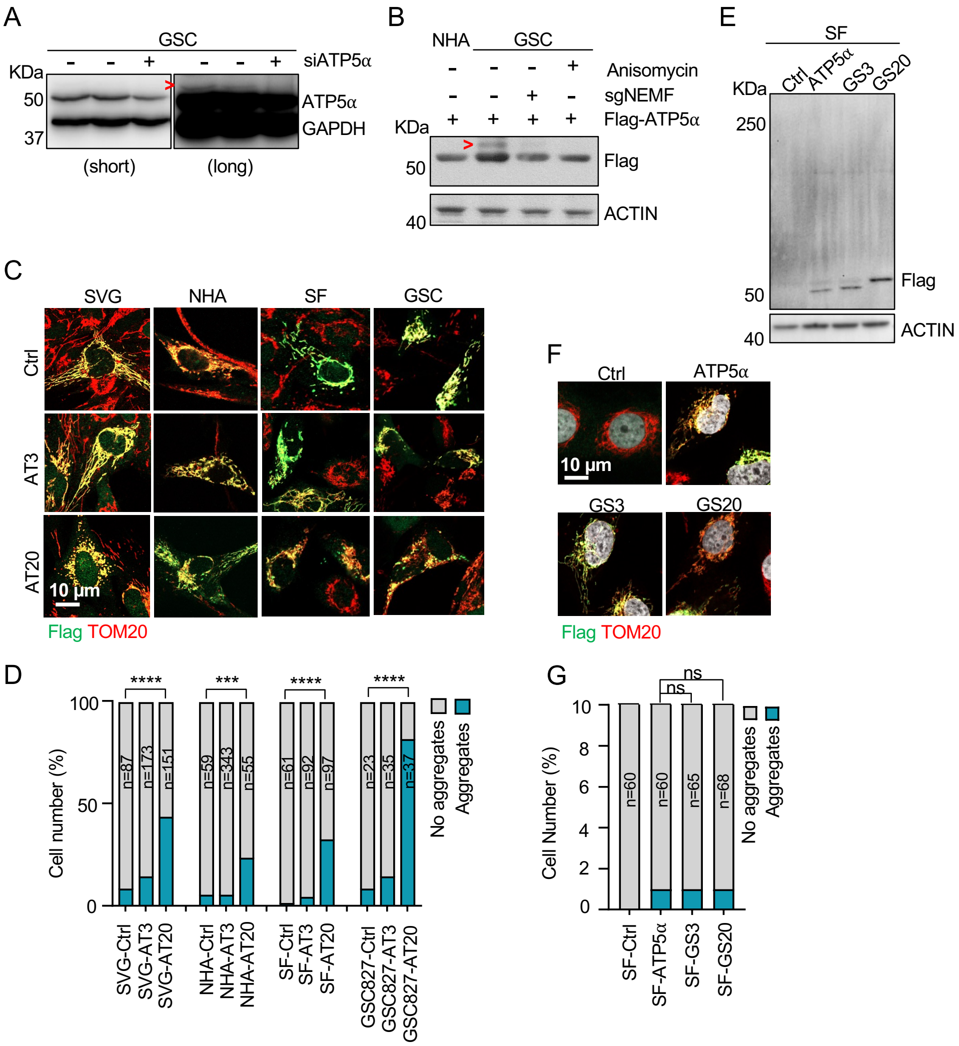

A
GSC
siATP5α
KDa
50
37
ATP5α
GAPDH
(short)
(long)
B
NHA
GSC
Anisomycin
sgNEMF
Flag-ATP5α
KDa
50
40
Flag
ACTIN
E
SF
KDa
Ctrl
ATP5α
GS3
GS20
250
50
40
Flag
ACTIN
C
SVG
NHA
SF
GSC
Ctrl
AT3
AT20
10 µm
Flag TOM20
F
Ctrl
ATP5α
10 µm
GS3
GS20
Flag TOM20
D
Cell number (%)
100
50
0
No aggregates
Aggregates
SVG-Ctrl
SVG-AT3
SVG-AT20
NHA-Ctrl
NHA-AT3
NHA-AT20
SF-Ctrl
SF-AT3
SF-AT20
GSC827-Ctrl
GSC827-AT3
GSC827-AT20
G
Cell Number (%)
10
8
6
4
2
0
ns
No aggregates
Aggregates
SF-Ctrl
SF-ATP5α
SF-GS3
SF-GS20

## Figure S3

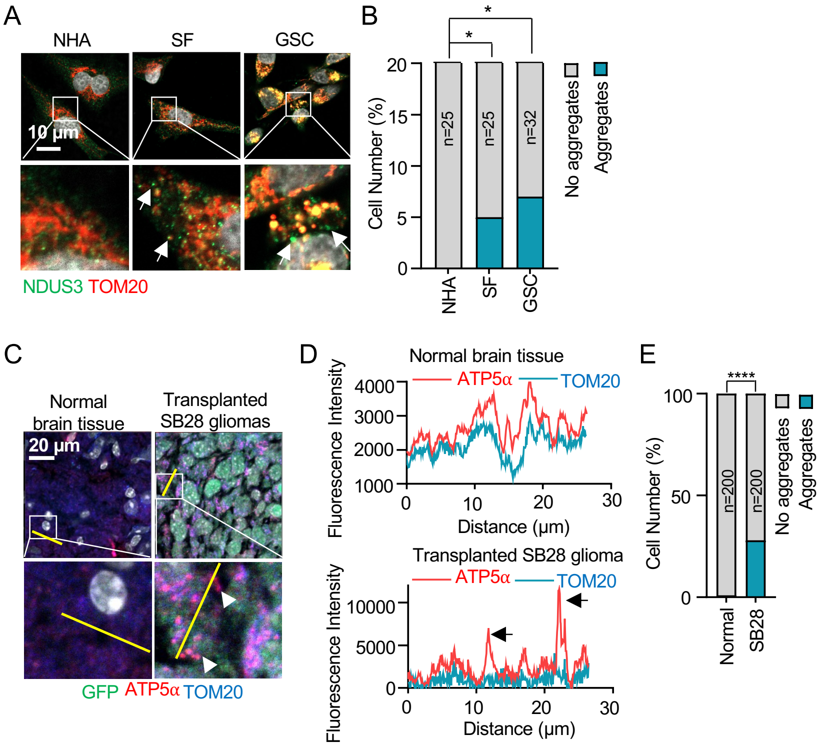
A
NHA
SF
GSC
10 μm
NDUS3 TOM20
B
Cell Number (%)
n=25
n=25
n=32
NHA
SF
GSC
No aggregates
Aggregates
C
Normal brain tissue
Transplanted SB28 gliomas
20 μm
GFP ATP5α TOM20
D
Normal brain tissue
ATP5α
TOM20
Fluorescence Intensity
Distance (μm)
Transplanted SB28 glioma
E
Cell Number (%)
n=200
n=200
Normal
SB28
No aggregates
Aggregates

# Figure S4

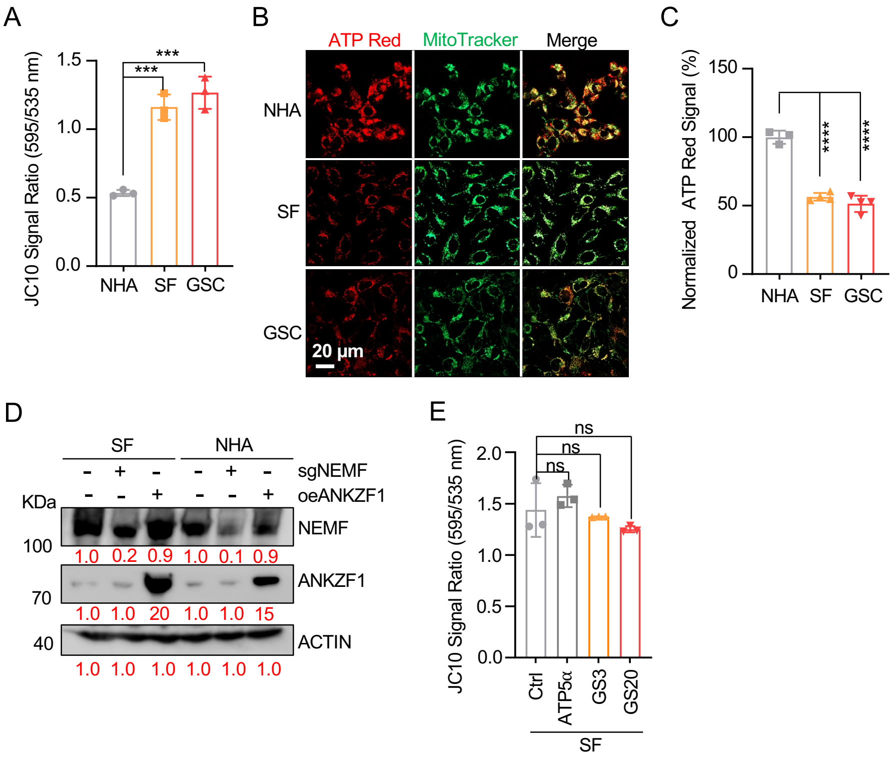
A
JC10 Signal Ratio (595/535 nm)
1.5
1.0
0.5
0.0
***
***
NHA
SF
GSC
B
ATP Red
MitoTracker
Merge
NHA
SF
GSC
20 µm
C
Normalized ATP Red Signal (%)
150
100
50
0
****
****
NHA
SF
GSC
D
SF
NHA
sgNEMF
oeANKZF1
- + - - + -
- - + - - +
KDa
100
70
40
NEMF
1.0 0.2 0.9 1.0 0.1 0.9
ANKZF1
1.0 1.0 20 1.0 1.0 15
ACTIN
1.0 1.0 1.0 1.0 1.0 1.0
E
JC10 Signal Ratio (595/535 nm)
2.0
1.5
1.0
0.5
0.0
ns
ns
ns
Ctrl
ATP5α
GS3
GS20
SF

## Figure S5

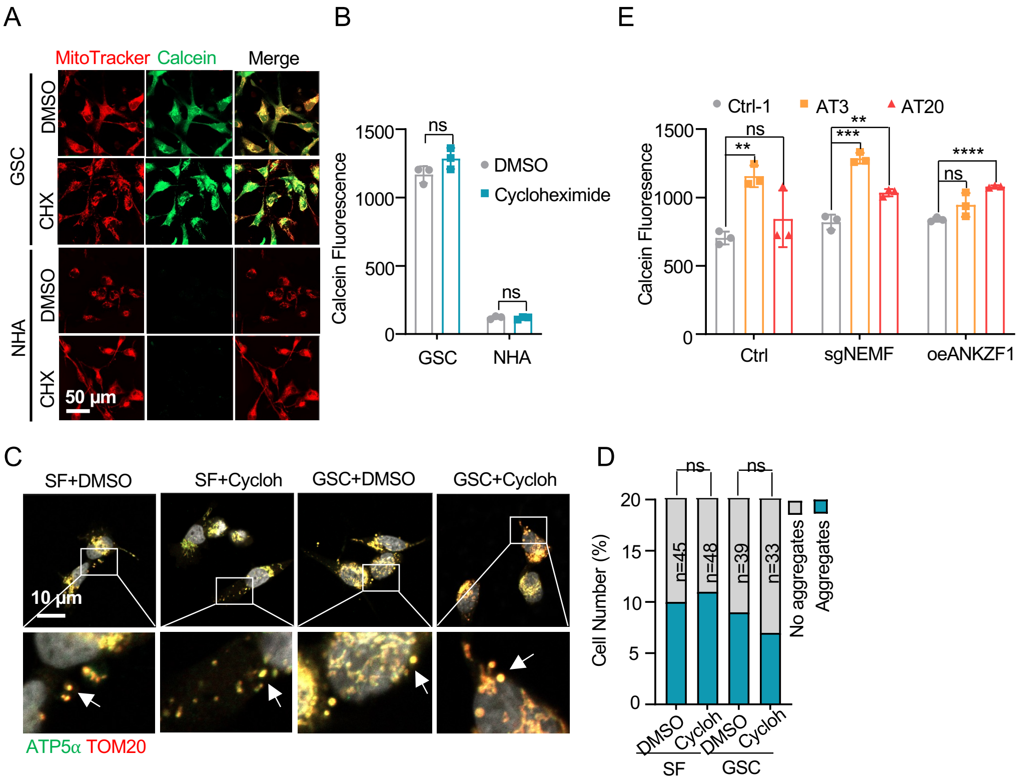

A
MitoTracker Calcein Merge
GSC
DMSO
CHX
NHA
DMSO
CHX
50 µm
B
ns
1500
1000
500
0
Calcein Fluorescence
GSC
NHA
DMSO
Cycloheximide
E
Ctrl-1
AT3
AT20
**
**
***
ns
ns
****
1500
1000
500
0
Calcein Fluoresence
Ctrl
sgNEMF
oeANKZF1
C
SF+DMSO
SF+Cycloh
GSC+DMSO
GSC+Cycloh
10 µm
ATP5α TOM20
D
ns
ns
20
15
10
5
0
Cell Number (%)
n=45
n=48
n=39
n=33
No aggregates
Aggregates
DMSO
Cycloh
DMSO
Cycloh
SF
GSC

## Figure S6

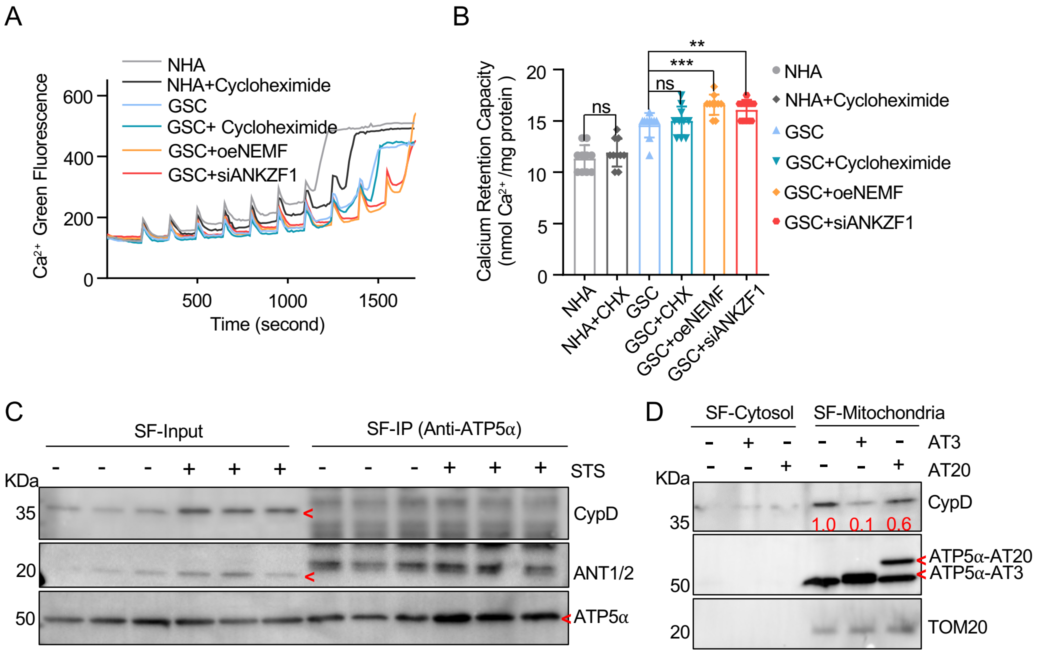
A
NHA
NHA+Cycloheximide
GSC
GSC+ Cycloheximide
GSC+oeNEMF
GSC+siANKZF1
Ca2+ Green Fluorescence
600
400
200
0
500
1000
1500
Time (second)
B
Calcium Retention Capacity (nmol Ca2+ /mg protein )
20
15
10
5
0
ns
ns
***
**
NHA
NHA+CHX
GSC
GSC+CHX
GSC+oeNEMF
GSC+siANKZF1
NHA
NHA+Cycloheximide
GSC
GSC+Cycloheximide
GSC+oeNEMF
GSC+siANKZF1
C
SF-Input
SF-IP (Anti-ATP5α)
STS
KDa
35
20
50
CypD
ANT1/2
ATP5α
D
SF-Cytosol
SF-Mitochondria
AT3
AT20
KDa
35
50
20
CypD
1.0
0.1
0.6
ATP5α-AT20
ATP5α-AT3
TOM20

## Figure S7

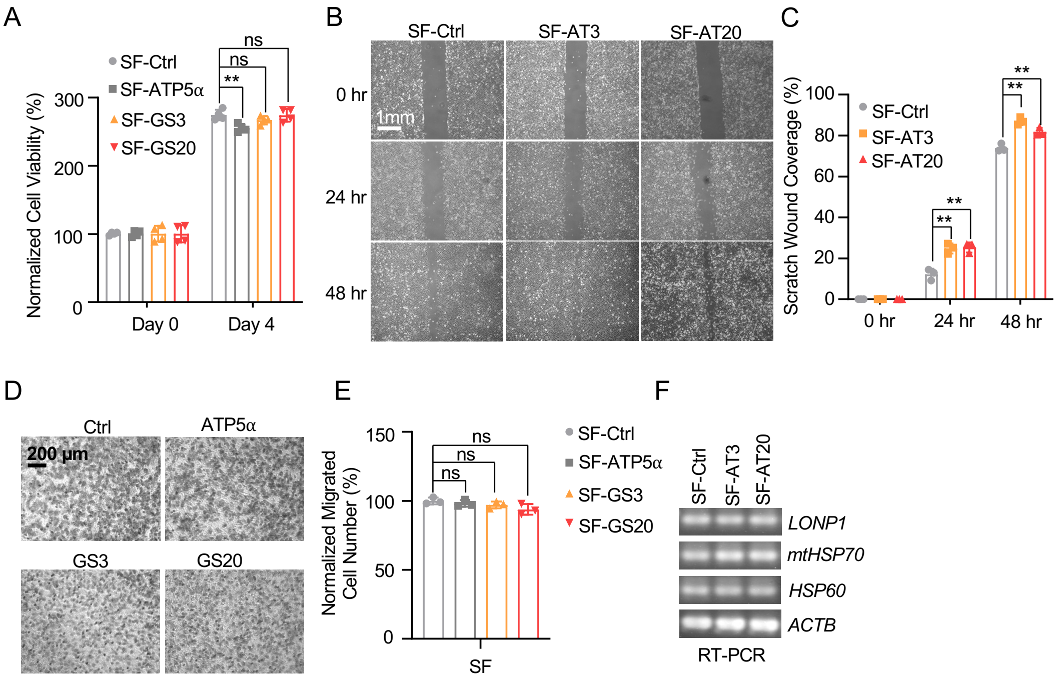
A
SF-Ctrl
SF-ATP5α
SF-GS3
SF-GS20
Normalized Cell Viability (%)
300
200
100
0
Day 0
Day 4
ns
ns
**
B
SF-Ctrl
SF-AT3
SF-AT20
0 hr
24 hr
48 hr
1mm
C
Scratch Wound Coverage (%)
100
80
60
40
20
0
SF-Ctrl
SF-AT3
SF-AT20
0 hr
24 hr
48 hr
**
D
Ctrl
ATP5α
200 μm
GS3
GS20
E
Normalized Migrated Cell Number (%)
150
100
50
0
SF
ns
SF-Ctrl
SF-ATP5α
SF-GS3
SF-GS20
F
SF-Ctrl
SF-AT3
SF-AT20
LONP1
mtHSP70
HSP60
ACTB
RT-PCR

## Figure S8

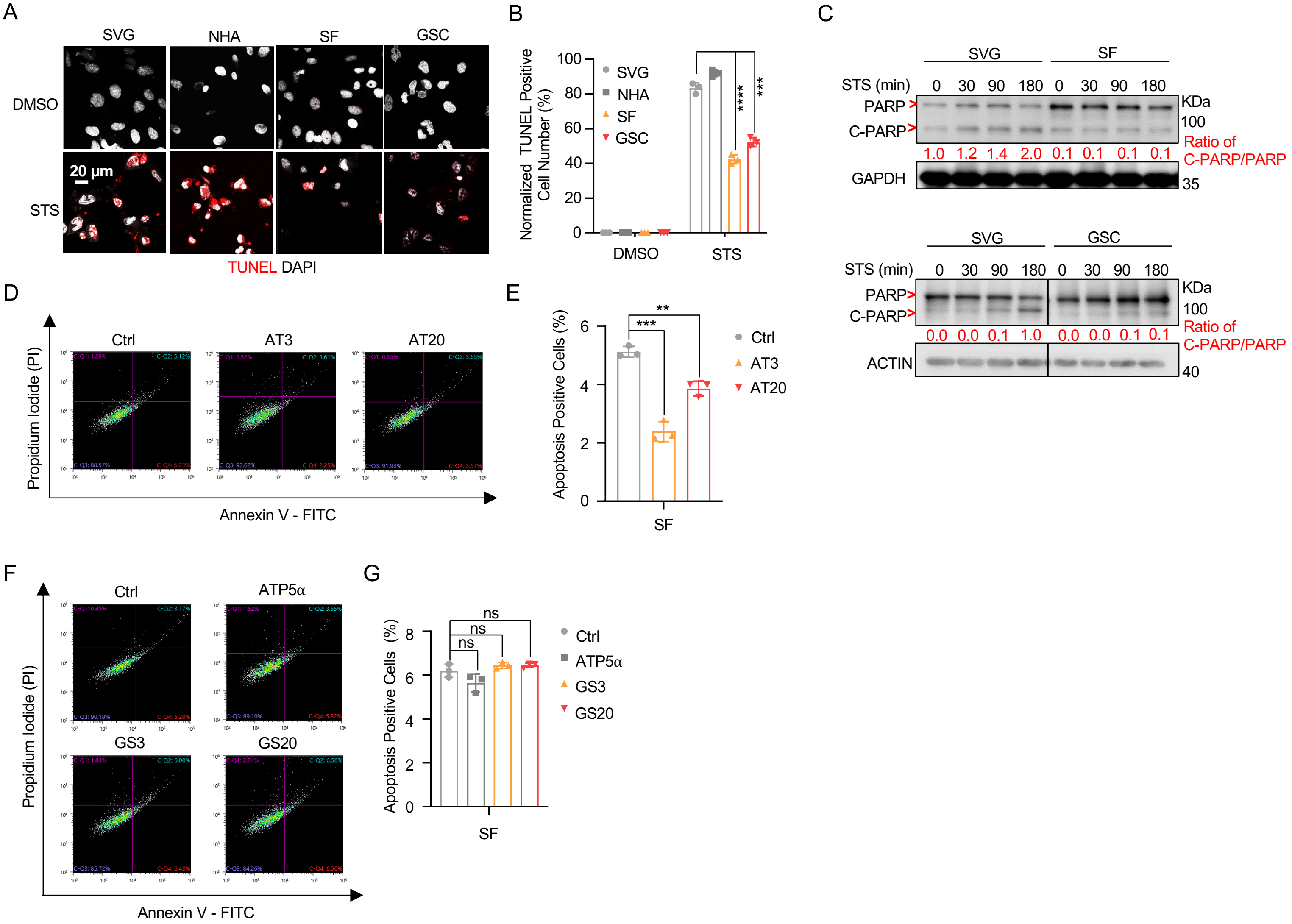
A
SVG
NHA
SF
GSC
DMSO
STS
20 μm
TUNEL DAPI
B
Normalized TUNEL Positive Cell Number (%)
SVG
NHA
SF
GSC
DMSO
STS
****
***
C
SVG
SF
STS (min)
0 30 90 180 0 30 90 180
PARP
C-PARP
KDa
100
Ratio of C-PARP/PARP
1.0 1.2 1.4 2.0 0.1 0.1 0.1 0.1
GAPDH
35
SVG
GSC
STS (min)
0 30 90 180 0 30 90 180
PARP
C-PARP
KDa
100
Ratio of C-PARP/PARP
0.0 0.0 0.1 1.0 0.0 0.0 0.1 0.1
ACTIN
40
D
Propidium Iodide (PI)
Ctrl
AT3
AT20
Annexin V - FITC
E
Apoptosis Positive Cells (%)
Ctrl
AT3
AT20
SF
***
**
F
Propidium Iodide (PI)
Ctrl
ATP5α
GS3
GS20
Annexin V - FITC
G
Apoptosis Positive Cells (%)
Ctrl
ATP5α
GS3
GS20
ns
SF

**Figure S9**

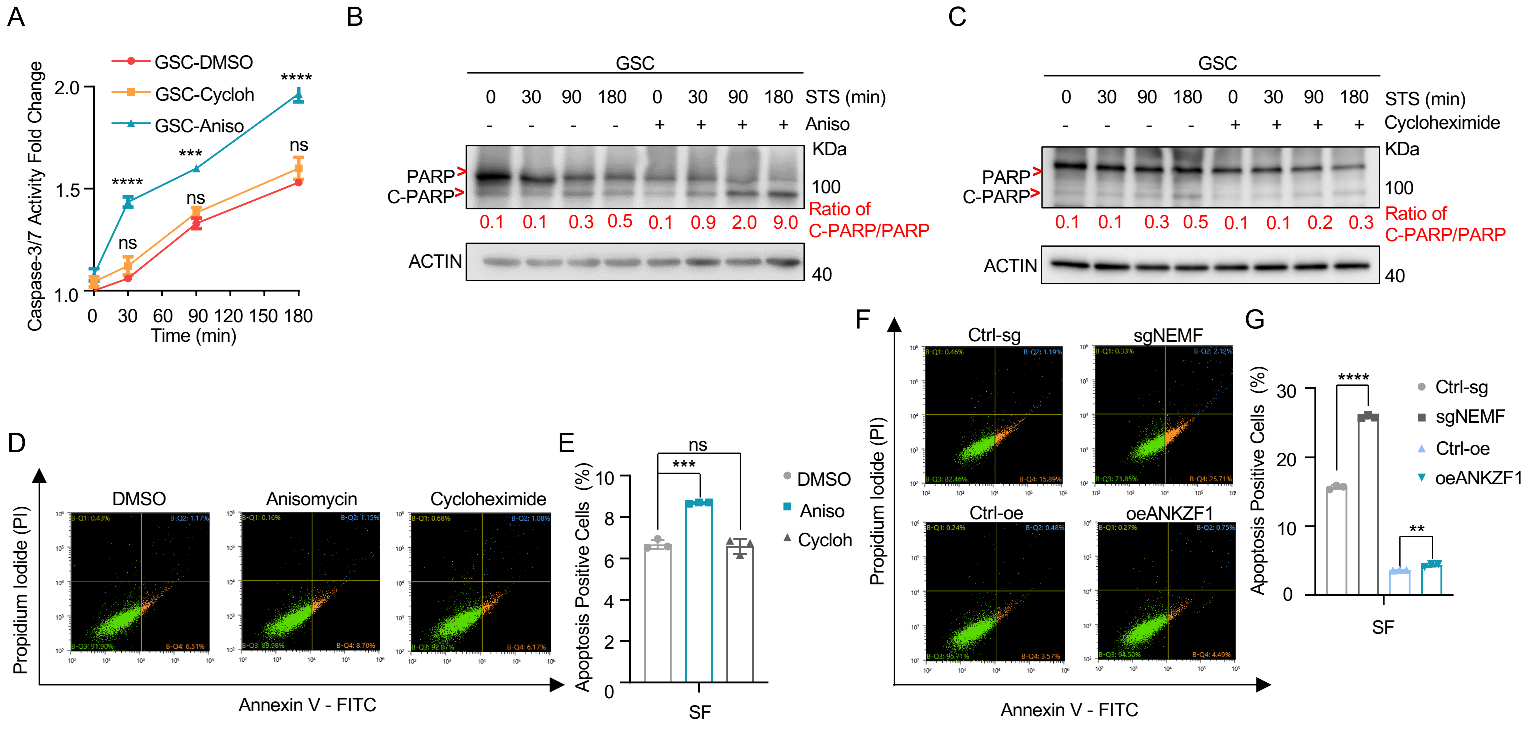

A
GSC-DMSO
GSC-Cycloh
GSC-Aniso
Caspase-3/7 Activity Fold Change
Time (min)
B
GSC
STS (min)
Aniso
PARP
C-PARP
KDa
Ratio of C-PARP/PARP
ACTIN
C
GSC
STS (min)
Cycloheximide
PARP
C-PARP
KDa
Ratio of C-PARP/PARP
ACTIN
D
DMSO
Anisomycin
Cycloheximide
Propidium Iodide (PI)
Annexin V - FITC
E
Apoptosis Positive Cells (%)
DMSO
Aniso
Cycloh
SF
F
Ctrl-sg
sgNEMF
Ctrl-oe
oeANKZF1
Propidium Iodide (PI)
Annexin V - FITC
G
Apoptosis Positive Cells (%)
Ctrl-sg
sgNEMF
Ctrl-oe
oeANKZF1
SF

## Table S1

**Table S1. Differential expression analysis for RQC genes in GBM patients and healthy controls**

| Gene | logFC | AveExpr | t | P.Value | adj.P.Val |
|---|---|---|---|---|---|
| RACK1 ⬆ | 2.224548565 | 9.048113333 | 19.24641934 | 2.62E-57 | 6.69E-56 |
| ASCC3 ⬆ | 1.738216567 | 2.717246389 | 19.27211713 | 2.05E-57 | 5.27E-56 |
| ASCC1 ⬆ | 1.689584768 | 4.257915556 | 17.8774401 | 1.20E-51 | 2.18E-50 |
| ASCC2 ⬆ | 1.471467207 | 4.153399167 | 15.9118075 | 1.43E-43 | 1.68E-42 |
| ABCE1 ⬆ | 1.32826428 | 3.81695 | 13.79248369 | 4.69E-35 | 3.60E-34 |
| VCP ⬆ | 1.050066326 | 6.321021667 | 9.828944092 | 2.33E-20 | 9.31E-20 |
| GIGYF2 | 0.985695112 | 3.786421389 | 10.25440005 | 8.00E-22 | 3.41E-21 |
| MAP3K20 | 0.962218073 | 1.863793889 | 8.711292467 | 1.10E-16 | 3.75E-16 |
| PELO | 0.92860628 | 2.2885625 | 10.60122263 | 4.85E-23 | 2.18E-22 |
| KLHDC10 | 0.854921284 | 3.492322222 | 9.064976509 | 8.08E-18 | 2.88E-17 |
| EDF1 | 0.82091202 | 8.444505 | 7.278600017 | 2.12E-12 | 5.94E-12 |
| XRN1 | 0.809119864 | 1.518371111 | 8.54020086 | 3.80E-16 | 1.26E-15 |
| LTN1 | 0.786409776 | 1.9716 | 9.962815742 | 8.14E-21 | 3.32E-20 |
| MKRN1 | 0.769369764 | 5.745359444 | 7.400071791 | 9.65E-13 | 2.74E-12 |
| RCHY1 | 0.652647968 | 4.276126111 | 6.840213538 | 3.40E-11 | 8.93E-11 |
| ZNF598 | 0.62380412 | 4.006663611 | 6.043582081 | 3.76E-09 | 8.90E-09 |
| HBS1L | 0.291107388 | 4.701389722 | 2.72853772 | 0.006672805 | 0.010370549 |
| NEMF | 0.194631373 | 4.566962778 | 2.575063266 | 0.010419695 | 0.015855894 |
| ANKZF1 ⬇ | -0.436070986 | 4.620298333 | -3.65005718 | 0.000300886 | 0.000525859 |

Abbreviations: RQC, ribosome-associated quality control; GBM, glioblastoma.
