# Supplementary material for: Mitochondrial stress-induced protein carboxyl-terminal alanine threonine tailing (msiCAT-tailing) facilitates glioblastoma tumorigenesis through the modulation of mitochondrial functions
Source: bioRxiv. 2025 Jun 16:2024.05.15.594447. Originally published 2024 May 18. Preprint. [Version 2] doi: 10.1101/2024.05.15.594447 (PMC11118334; doi:10.1101/2024.05.15.594447)
Supplement: Supplementary file 1 [file media-1.pdf]

Figure S1

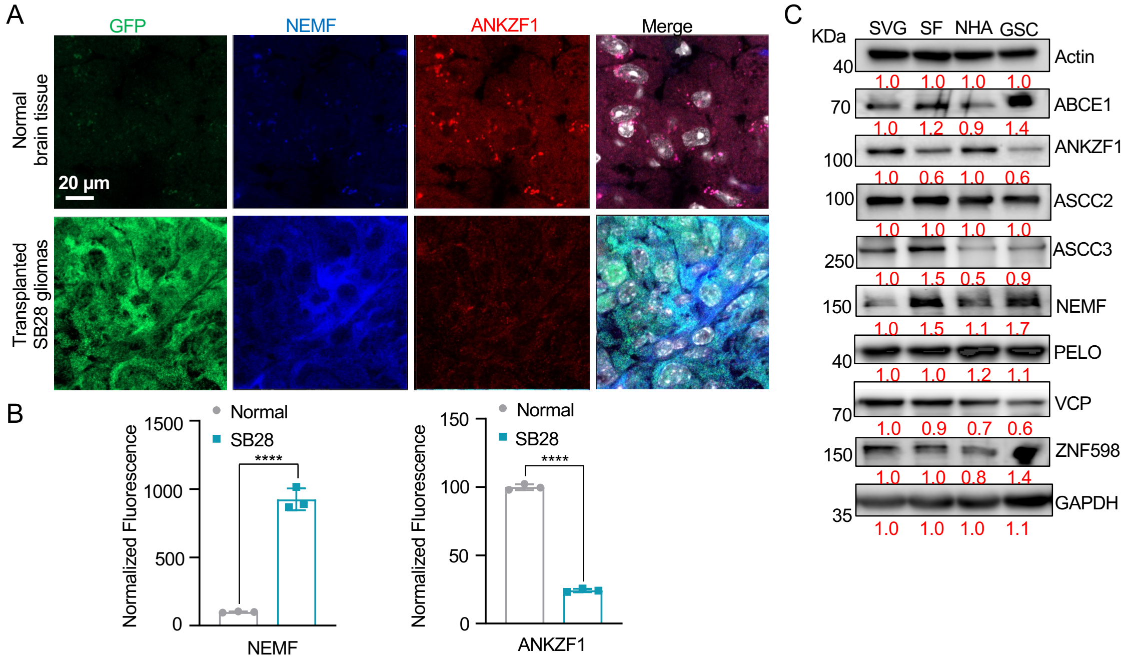
A
GFP
NEMF
ANKZF1
Merge
Normal brain tissue
Transplanted SB28 gliomas
20 μm
B
Normalized Fluorescence
Normal
SB28
****
1500
1000
500
0
NEMF
150
100
50
0
ANKZF1
C
KDa
SVG
SF
NHA
GSC
Actin
40
1.0 1.0 1.0 1.0
ABCE1
70
1.0 1.2 0.9 1.4
ANKZF1
100
1.0 0.6 1.0 0.6
ASCC2
100
1.0 1.0 1.0 1.0
ASCC3
250
1.0 1.5 0.5 0.9
NEMF
150
1.0 1.5 1.1 1.7
PELO
40
1.0 1.0 1.2 1.1
VCP
70
1.0 0.9 0.7 0.6
ZNF598
150
1.0 1.0 0.8 1.4
GAPDH
35
1.0 1.0 1.0 1.1

Figure S2

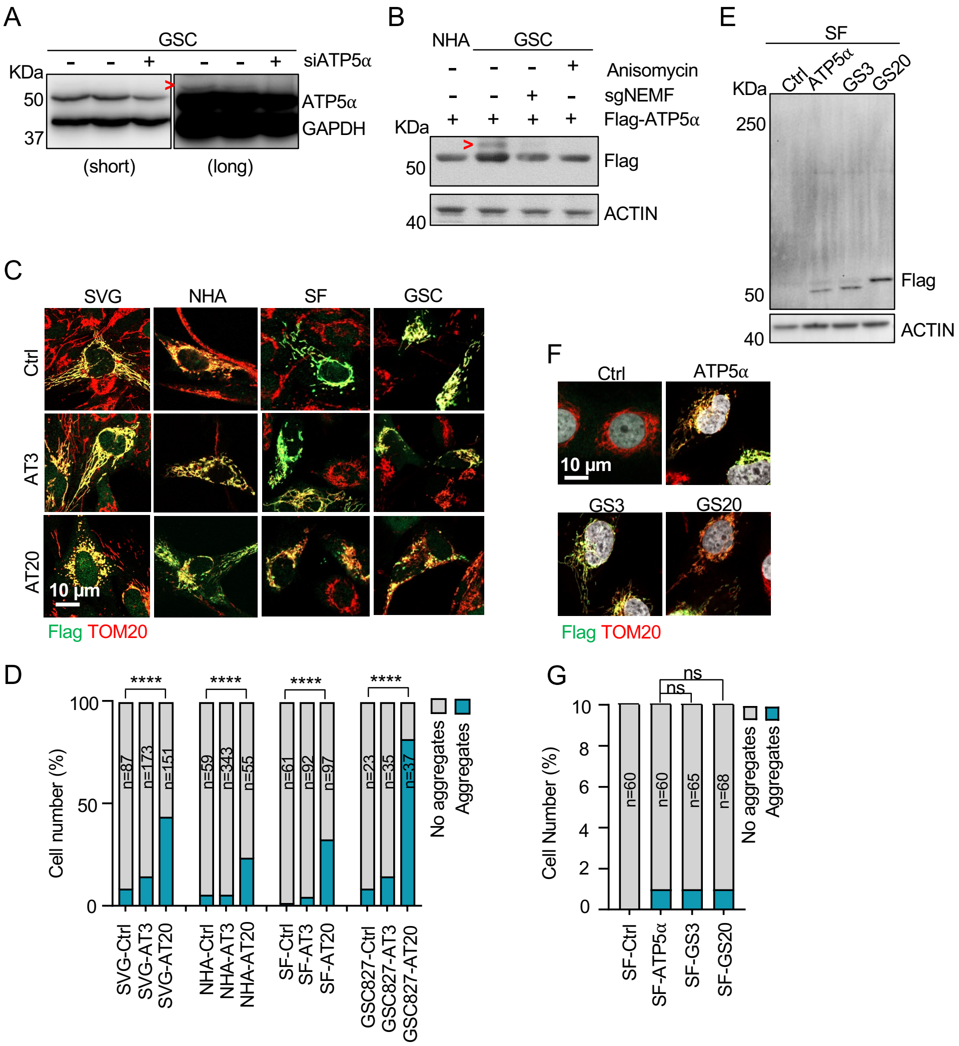
A
GSC
siATP5α
KDa
50
37
ATP5α
GAPDH
(short)
(long)
B
NHA
GSC
Anisomycin
sgNEMF
Flag-ATP5α
KDa
50
40
Flag
ACTIN
E
SF
Ctrl
ATP5α
GS3
GS20
KDa
250
50
40
Flag
ACTIN
C
SVG
NHA
SF
GSC
Ctrl
AT3
AT20
10 μm
Flag TOM20
F
Ctrl
ATP5α
10 μm
GS3
GS20
Flag TOM20
D
Cell number (%)
****
No aggregates
Aggregates
n=87
n=173
n=151
n=59
n=343
n=55
n=61
n=92
n=97
n=23
n=35
n=37
SVG-Ctrl
SVG-AT3
SVG-AT20
NHA-Ctrl
NHA-AT3
NHA-AT20
SF-Ctrl
SF-AT3
SF-AT20
GSC827-Ctrl
GSC827-AT3
GSC827-AT20
G
Cell Number (%)
ns
n=60
n=60
n=65
n=68
No aggregates
Aggregates
SF-Ctrl
SF-ATP5α
SF-GS3
SF-GS20

Figure S3

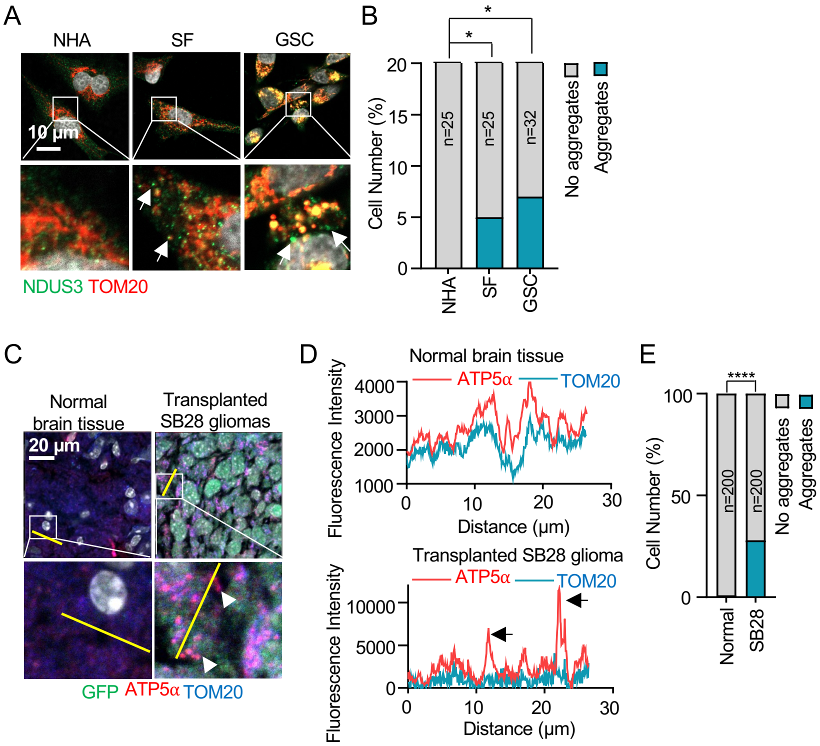

A
NHA
SF
GSC
10 μm
NDUS3 TOM20
B
Cell Number (%)
n=25
n=25
n=32
NHA
SF
GSC
No aggregates
Aggregates
C
Normal brain tissue
Transplanted SB28 gliomas
20 μm
GFP ATP5α TOM20
D
Normal brain tissue
ATP5α
TOM20
Fluorescence Intensity
Distance (μm)
Transplanted SB28 glioma
E
Cell Number (%)
n=200
n=200
Normal
SB28
No aggregates
Aggregates

Figure S4

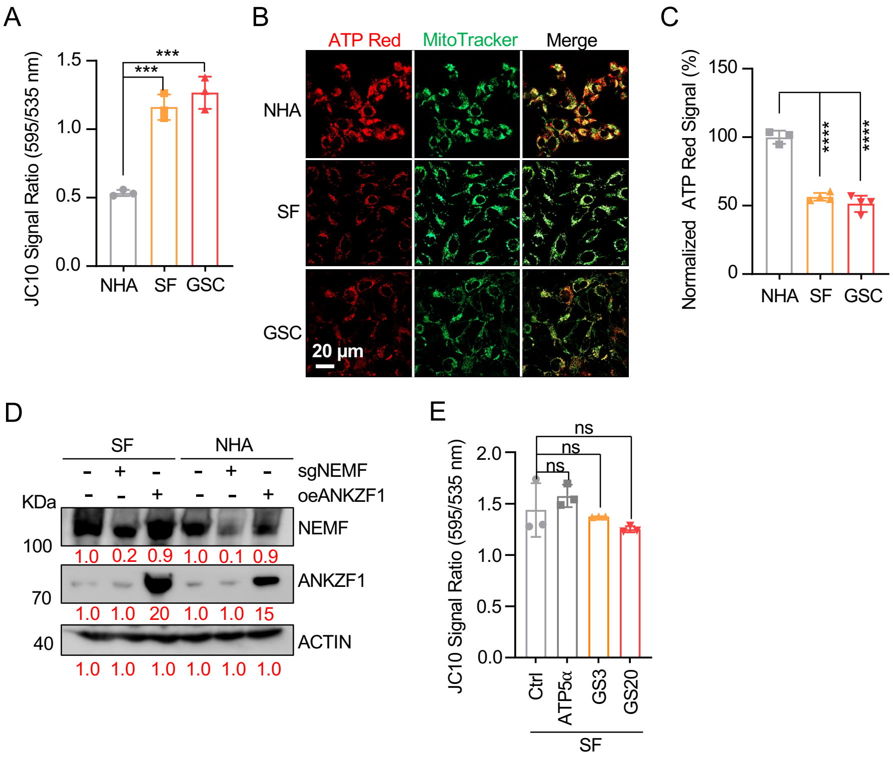

A
JC10 Signal Ratio (595/535 nm)
1.5
1.0
0.5
0.0
***
***
NHA
SF
GSC
B
ATP Red
MitoTracker
Merge
NHA
SF
GSC
20 µm
C
Normalized ATP Red Signal (%)
150
100
50
0
****
****
NHA
SF
GSC
D
SF
NHA
sgNEMF
oeANKZF1
KDa
NEMF
100
1.0 0.2 0.9 1.0 0.1 0.9
ANKZF1
70
1.0 1.0 20 1.0 1.0 15
ACTIN
40
1.0 1.0 1.0 1.0 1.0 1.0
E
JC10 Signal Ratio (595/535 nm)
2.0
1.5
1.0
0.5
0.0
ns
ns
ns
Ctrl
ATP5α
GS3
GS20
SF

Figure S5

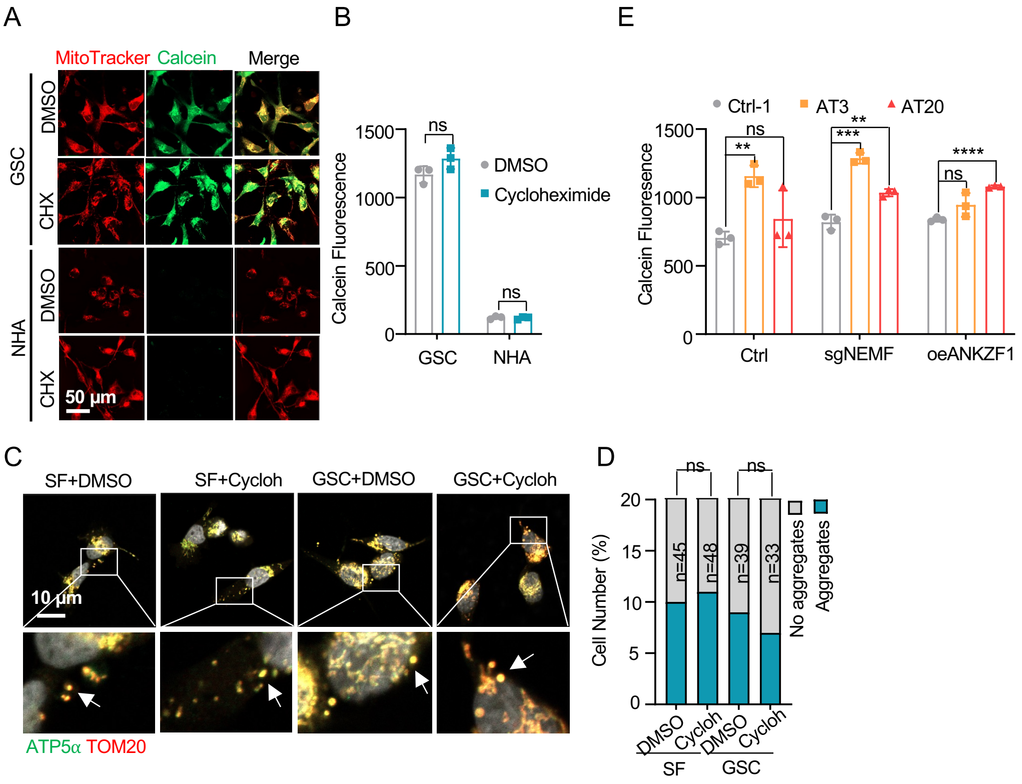

A
MitoTracker
Calcein
Merge
GSC
DMSO
CHX
NHA
DMSO
CHX
50 µm
B
Calcein Fluorescence
1500
1000
500
0
ns
ns
DMSO
Cycloheximide
GSC
NHA
E
Ctrl-1
AT3
AT20
Calcein Fluoresence
1500
1000
500
0
ns
**
***
**
ns
****
Ctrl
sgNEMF
oeANKZF1
C
SF+DMSO
SF+Cycloh
GSC+DMSO
GSC+Cycloh
10 µm
ATP5α
TOM20
D
Cell Number (%)
20
15
10
5
0
ns
ns
n=45
n=48
n=39
n=33
No aggregates
Aggregates
DMSO
Cycloh
DMSO
Cycloh
SF
GSC

Figure S6

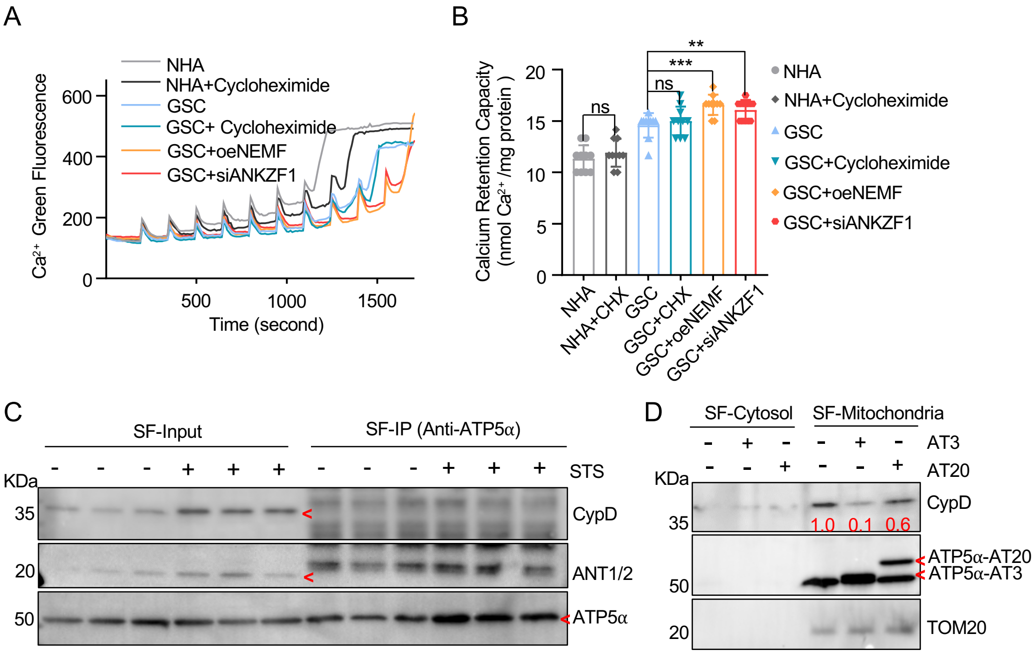
A
NHA
NHA+Cycloheximide
GSC
GSC+ Cycloheximide
GSC+oeNEMF
GSC+siANKZF1
Ca2+ Green Fluorescence
Time (second)
B
Calcium Retention Capacity (nmol Ca2+ /mg protein)
ns
***
**
NHA
NHA+CHX
GSC
GSC+CHX
GSC+oeNEMF
GSC+siANKZF1
NHA
NHA+Cycloheximide
GSC
GSC+Cycloheximide
GSC+oeNEMF
GSC+siANKZF1
C
SF-Input
SF-IP (Anti-ATP5α)
STS
KDa
CypD
ANT1/2
ATP5α
D
SF-Cytosol
SF-Mitochondria
AT3
AT20
KDa
CypD
1.0
0.1
0.6
ATP5α-AT20
ATP5α-AT3
TOM20

Figure S7

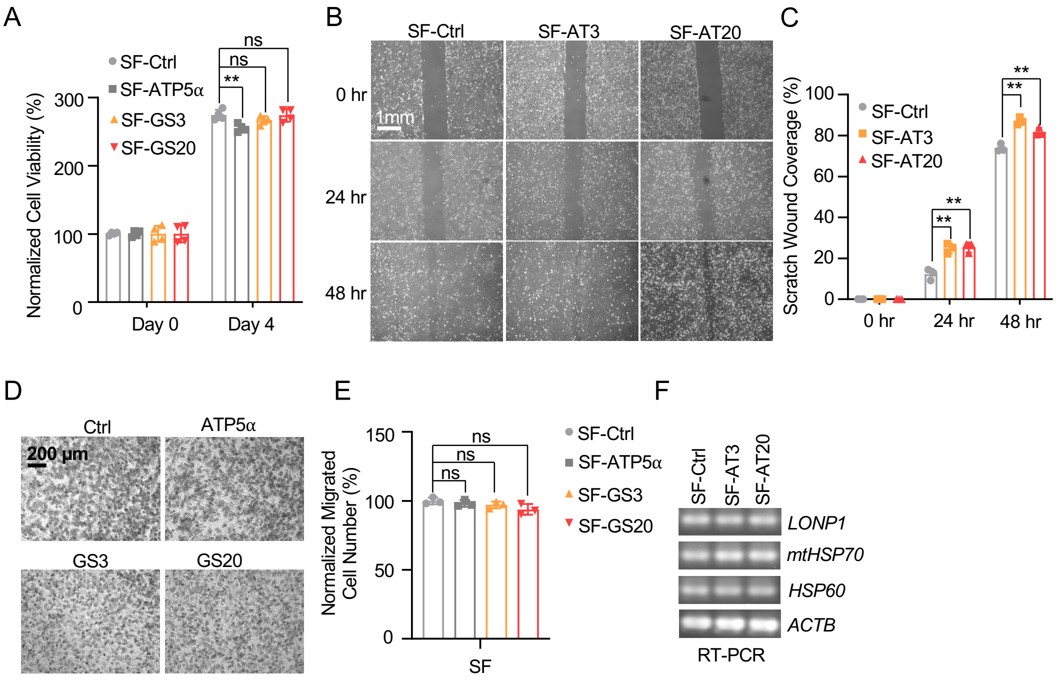

A
SF-Ctrl
SF-ATP5α
SF-GS3
SF-GS20
Normalized Cell Viability (%)
300
200
100
0
ns
ns
**
Day 0
Day 4
B
SF-Ctrl
SF-AT3
SF-AT20
0 hr
24 hr
48 hr
1mm
C
Scratch Wound Coverage (%)
100
80
60
40
20
0
SF-Ctrl
SF-AT3
SF-AT20
**
**
**
**
0 hr
24 hr
48 hr
D
Ctrl
ATP5α
200 μm
GS3
GS20
E
Normalized Migrated Cell Number (%)
150
100
50
0
ns
ns
ns
SF
SF-Ctrl
SF-ATP5α
SF-GS3
SF-GS20
F
SF-Ctrl
SF-AT3
SF-AT20
LONP1
mtHSP70
HSP60
ACTB
RT-PCR

Figure S8

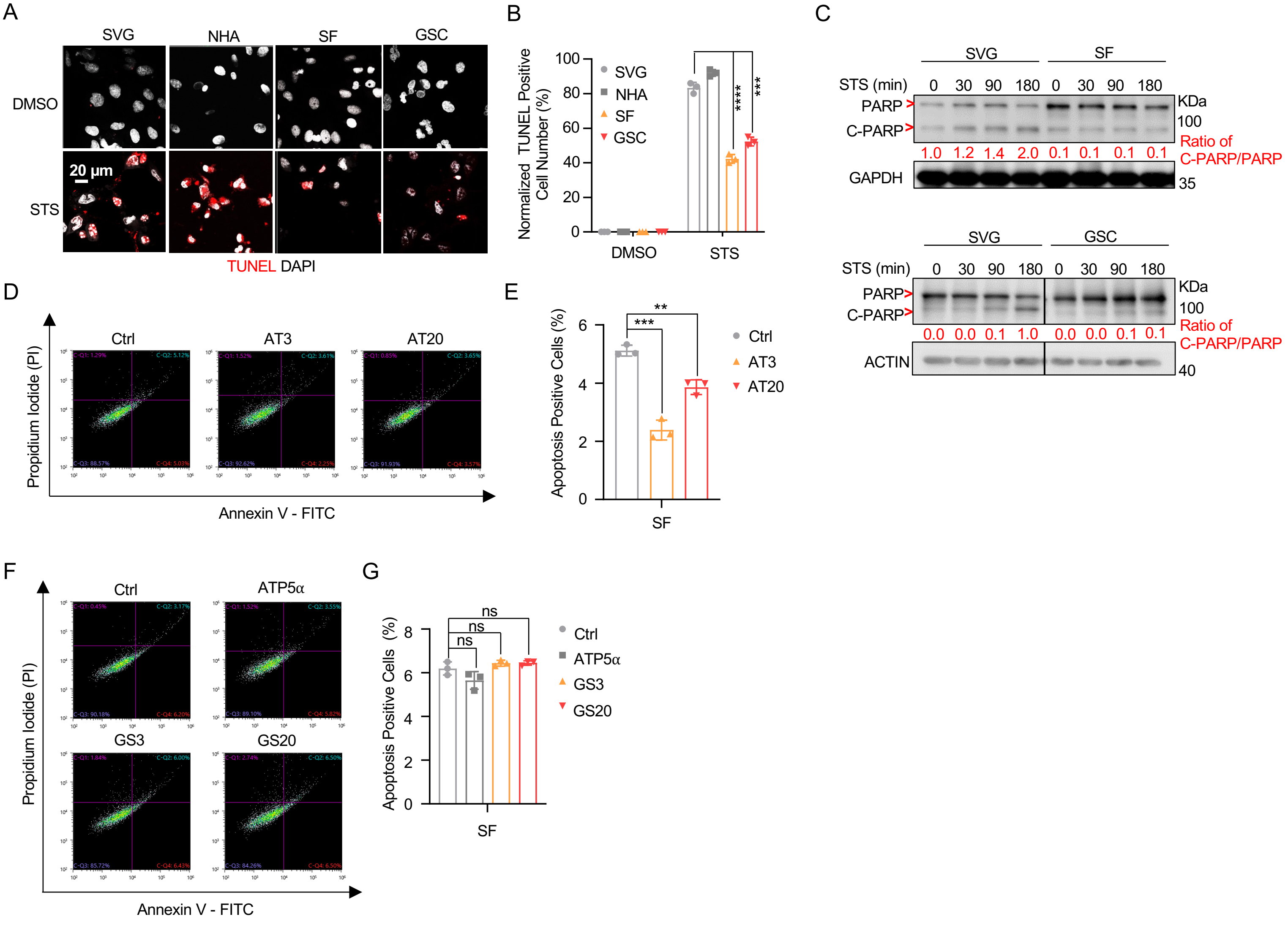

A
SVG
NHA
SF
GSC
DMSO
STS
20 µm
TUNEL DAPI
B
Normalized TUNEL Positive Cell Number (%)
100
80
60
40
20
0
SVG
NHA
SF
GSC
DMSO
STS
****
***
C
SVG
SF
STS (min)
0 30 90 180 0 30 90 180
PARP
C-PARP
GAPDH
KDa
100
35
Ratio of C-PARP/PARP
1.0 1.2 1.4 2.0 0.1 0.1 0.1 0.1
SVG
GSC
STS (min)
0 30 90 180 0 30 90 180
PARP
C-PARP
ACTIN
KDa
100
40
Ratio of C-PARP/PARP
0.0 0.0 0.1 1.0 0.0 0.0 0.1 0.1
D
Ctrl
AT3
AT20
Propidium Iodide (PI)
Annexin V - FITC
E
Apoptosis Positive Cells (%)
6
4
2
0
***
**
Ctrl
AT3
AT20
SF
F
Ctrl
ATP5α
GS3
GS20
Propidium Iodide (PI)
Annexin V - FITC
G
Apoptosis Positive Cells (%)
8
6
4
2
0
ns
ns
ns
Ctrl
ATP5α
GS3
GS20
SF

Figure S9

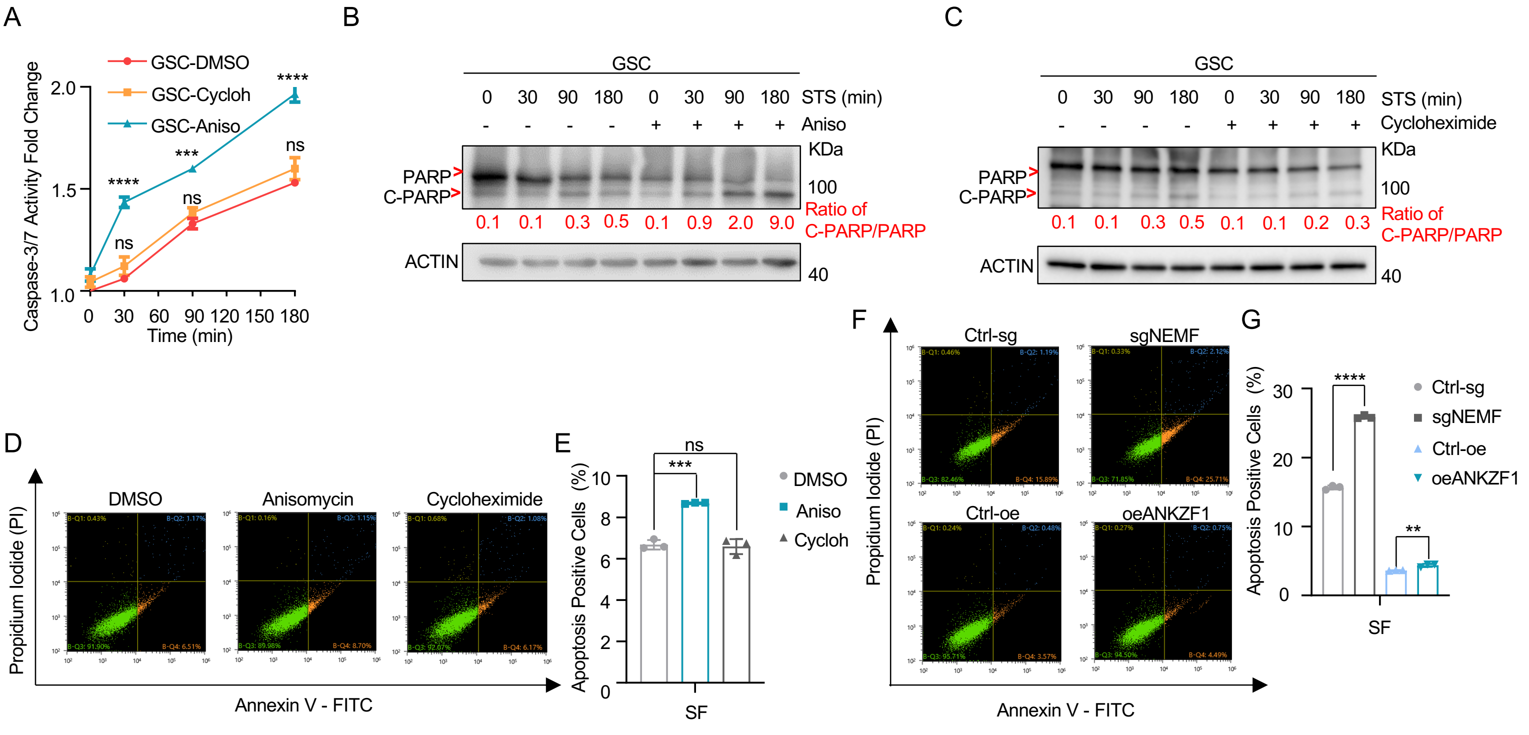
A
GSC-DMSO
GSC-Cycloh
GSC-Aniso
Caspase-3/7 Activity Fold Change
Time (min)
B
GSC
STS (min)
Aniso
KDa
PARP
C-PARP
Ratio of C-PARP/PARP
0.1 0.1 0.3 0.5 0.1 0.9 2.0 9.0
ACTIN
C
GSC
STS (min)
Cycloheximide
KDa
PARP
C-PARP
Ratio of C-PARP/PARP
0.1 0.1 0.3 0.5 0.1 0.1 0.2 0.3
ACTIN
D
DMSO
Anisomycin
Cycloheximide
Propidium Iodide (PI)
Annexin V - FITC
E
Apoptosis Positive Cells (%)
DMSO
Aniso
Cycloh
SF
F
Ctrl-sg
sgNEMF
Ctrl-oe
oeANKZF1
Propidium Iodide (PI)
Annexin V - FITC
G
Apoptosis Positive Cells (%)
Ctrl-sg
sgNEMF
Ctrl-oe
oeANKZF1
SF

Table S1

Table S1. Differential expression analysis for RQC genes in GBM patients and healthy controls

Gene	logFC	AveExpr	t	P.Value	adj.P.Val
RACK1 ↑	2.224548565	9.048113333	19.24641934	2.62E-57	6.69E-56
ASCC3 ↑	1.738216567	2.717246389	19.27211713	2.05E-57	5.27E-56
ASCC1 ↑	1.689584768	4.257915556	17.8774401	1.20E-51	2.18E-50
ASCC2 ↑	1.471467207	4.153399167	15.9118075	1.43E-43	1.68E-42
ABCE1 ↑	1.32826428	3.81695	13.79248369	4.69E-35	3.60E-34
VCP ↑	1.050066326	6.321021667	9.828944092	2.33E-20	9.31E-20
GIGYF2	0.985695112	3.786421389	10.25440005	8.00E-22	3.41E-21
MAP3K20	0.962218073	1.863793889	8.711292467	1.10E-16	3.75E-16
PELO	0.92860628	2.2885625	10.60122263	4.85E-23	2.18E-22
KLHDC10	0.854921284	3.492322222	9.064976509	8.08E-18	2.88E-17
EDF1	0.82091202	8.444505	7.278600017	2.12E-12	5.94E-12
XRN1	0.809119864	1.518371111	8.54020086	3.80E-16	1.26E-15
LTN1	0.786409776	1.9716	9.962815742	8.14E-21	3.32E-20
MKRN1	0.769369764	5.745359444	7.400071791	9.65E-13	2.74E-12
RCHY1	0.652647968	4.276126111	6.840213538	3.40E-11	8.93E-11
ZNF598	0.62380412	4.006663611	6.043582081	3.76E-09	8.90E-09
HBS1L	0.291107388	4.701389722	2.72853772	0.006672805	0.010370549
NEMF	0.194631373	4.566962778	2.575063266	0.010419695	0.015855894
ANKZF1 ↓	-0.436070986	4.620298333	-3.65005718	0.000300886	0.000525859

Abbreviations: RQC, ribosome-associated quality control; GBM, glioblastoma.
